# Patterns of Linkage Disequilibrium and Long Range Hitchhiking in Evolving Experimental *Drosophila melanogaster* Populations

**DOI:** 10.1093/molbev/msu320

**Published:** 2014-11-17

**Authors:** Susanne U. Franssen, Viola Nolte, Ray Tobler, Christian Schlötterer

**Affiliations:** ^1^Institut für Populationsgenetik, Vetmeduni Vienna, Vienna, Austria

**Keywords:** experimental evolution, haplotype sequencing, long range genetic hitchhiking, time series, standing genetic variation, selection on rare variants

## Abstract

Whole-genome resequencing of experimental populations evolving under a specific selection regime has become a popular approach to determine genotype–phenotype maps and understand adaptation to new environments. Despite its conceptual appeal and success in identifying some causative genes, it has become apparent that many studies suffer from an excess of candidate loci. Several explanations have been proposed for this phenomenon, but it is clear that information about the linkage structure during such experiments is needed. Until now only Pool-Seq (whole-genome sequencing of pools of individuals) data were available, which do not provide sufficient information about the correlation between linked sites. We address this problem in two complementary analyses of three replicate *Drosophila melanogaster* populations evolving to a new hot temperature environment for almost 70 generations. In the first analysis, we sequenced 58 haploid genomes from the founder population and evolved flies at generation 67. We show that during the experiment linkage disequilibrium (LD) increased almost uniformly over much greater distances than typically seen in *Drosophila*. In the second analysis, Pool-Seq time series data of the three replicates were combined with haplotype information from the founder population to follow blocks of initial haplotypes over time. We identified 17 selected haplotype-blocks that started at low frequencies in the base population and increased in frequency during the experiment. The size of these haplotype-blocks ranged from 0.082 to 4.01 Mb. Moreover, between 42% and 46% of the top candidate single nucleotide polymorphisms from the comparison of founder and evolved populations fell into the genomic region covered by the haplotype-blocks. We conclude that LD in such rising haplotype-blocks results in long range hitchhiking over multiple kilobase-sized regions. LD in such haplotype-blocks is therefore a major factor contributing to an excess of candidate loci. Although modifications of the experimental design may help to reduce the hitchhiking effect and allow for more precise mapping of causative variants, we also note that such haplotype-blocks might be well suited to study the dynamics of selected genomic regions during experimental evolution studies.

## Introduction

Evolve and resequence studies (E&R; Turner et al. 2011; Schlötterer et al. 2014), the combination of experimental evolution and whole-genome sequencing of pooled individuals (Pool-Seq), hold great promise in identifying targets of selection on a genome-wide scale. Generally, E&R studies have been applied to address two major aims: 1) Determination the genotype–phenotype map often carried out by explicit truncating selection on a trait of interest (e.g., Burke et al. 2010; Turner et al. 2011) or 2) understanding the processes of adaption through laboratory natural selection operating on fitness in a new environment with no explicit link to a specific trait (e.g., Orozco-terWengel et al. 2012).

The ease of handling *Drosophila melanogaster* in the laboratory in combination with its relatively short generation time, high levels of polymorphism, and low levels of linkage disequilibrium (LD) (Mackay et al. 2012) has made *D. melanogaster* the most frequently used multicellular organism in E&R experiments. It has been used to study a broad range of traits, including developmental time (Burke et al. 2010), hypoxia tolerance (Zhou et al. 2011), body size (Turner et al. 2011), life span (Remolina et al. 2012), courtship song (Turner and Miller 2012), parasitoid resistance (Jalvingh et al. 2014), and resistance to *Drosophila* C virus (DCV) (Martins et al. 2014). Moreover, a few studies did not focus on a specific trait, but instead employed laboratory natural selection under different temperature regimes (Orozco-terWengel et al. 2012; Tobler et al. 2014) or different diets (Reed et al. 2014).

Adaptation in E&R studies is expected to occur through selection on favorable alleles segregating in the starting population. Hence, the precision with which targets of selection can be identified partly depends on the amount of LD in the starting population and the number of recombination events during the experiment. Computer simulations suggest that experimental evolution with large population sizes, a large number of founding haplotypes, and multiple replicate populations combined with moderately strong selection hold greatest promise to identify selected sites (Kofler and Schlötterer 2013; Baldwin-Brown et al. 2014; Kessner and Novembre 2014).

For a simple trait, such as DCV resistance, E&R has successfully identified functionally diverged genes (Martins et al. 2014). Most studies, however, detect more candidate loci than can be explained by selection on causative variants alone (Nuzhdin and Turner 2013). This excess of significant sites might be due to hitchhiking of neutral variants linked to selected sites. To date, however, all E&R studies with recombination during the experiment have sequenced pools of individuals, and thus the influence of linkage between selected and neutral sites could not be addressed directly. Orozco-terWengel et al. (2012) and Tobler et al. (2014) used an indirect approach to estimate the extent to which close linkage to selected sites may have inflated the number of candidate loci. Measuring the drop in allele frequency change of single nucleotide polymorphisms (SNPs) flanking candidate loci, they found that the effect of linkage was restricted to a small window of 400 bp around a candidate site. Thus, they concluded that short range LD could not explain the large number of candidates observed in their study. Tobler et al. (2014) also approached the question of nonindependence of candidates from a different angle. Comparing candidate SNPs of independent sets of replicate populations, they found concordance in top ranked candidates also for short intronic sites, good proxies for neutral loci (e.g., Clemente and Vogl 2012). In the absence of haplotype information, however, the authors could not determine which of a set of possible causes—namely low recombining regions, inversions, and strong selection on low frequency sites—contributed most to the observed nonindependence of candidate SNPs.

LD and/or haplotype information in E&R is required to address the effect of association between candidate SNPs. However, current methods for inferring LD from Pool-Seq data are limited to very short genomic regions (Feder et al. 2012). Similarly, methods to estimate haplotype frequencies from Pool-Seq data rely on complete information about the founder haplotypes and operate on a window size of fixed genomic length, for which recombination is assumed to be absent, whereas no phase information is obtained between subsequent windows (Long et al. 2011; harp (v14-09-25) Kessner et al. 2013; Burke et al. 2014). An additional method by Cubillos et al. (2013) estimates allele frequencies of all known founding haplotypes using marker SNPs in Pool-Seq data of evolved populations but similarly does not estimate linkage between neighboring sites in the evolved population. Unless an E&R study uses a starting population of already known haplotypes such as the Drosophila Genetic Reference Panel (e.g., as done in Turner and Miller 2012), it is extremely cost intensive to obtain complete haplotype information of founding haplotypes. We therefore pursued a different strategy that uses a subset of the founding haplotypes to study the dynamics of LD in *D. melanogaster* populations that evolve in a new temperature regime. Furthermore, our strategy is not limited to identify haplotype-blocks of a predetermined length. First, we sequenced full genome haplotypes for 29 of at least 113 founder haplotypes and 29 evolved haplotypes of generation 67 to analyze genome-wide changes in LD during experimental evolution. Second, by combining Pool-Seq of time series data from three replicate populations with the subset of 29 founder haplotypes, we estimate frequencies of founder haplotype-blocks that increase in frequency over time. Our results demonstrate how selection on low frequency variants results in long range hitchhiking and an excess of candidate SNPs. We conclude that 1) long range LD can play a profound role during laboratory selection experiments, 2) the effect of long range hitchhiking is stronger if selection acts on rare variants and existing LD, and 3) haplotype information from the starting population combined with time series data is crucial for identification of high confidence targets of selection when selection acts on rare variants.

## Results and Discussion

### Determination of Full Genome Haplotypes during Experimental Evolution

As a first step, we determined full genome sequences of 29 haplotypes of each the experimental starting population and a late evolved population at generation 67 to analyze genome-wide changes in LD during experimental evolution. We investigate to what extent changes in LD are related to several factors, such as chromosome, distance, initial LD, recombination rate, and inversions.

#### Chromosomal Haplotypes

To infer chromosomal haplotypes, we crossed individuals from the population of interest to a reference strain with a known genome sequence. After sequencing an F1 female, the haplotype was determined by subtracting the reference sequence from the F1 genotype (supplementary fig. S1, Supplementary Material online; see Materials and Methods). We determined 29 haplotypes from the founder population (base, b1–b29) and one population that has evolved for 67 generations (e1–e29) in a hot environment fluctuating between 18 and 28°C. Sequencing coverage across all 58 individuals was high, with a mean coverage of at least 35-fold for 50% of all F1 individuals (supplementary fig. S2, Supplementary Material online). This allowed for allele calling and haplotype phasing for more than 90% of the euchromatic genome in 23 (base) or 24 (hot evolved) individuals (supplementary fig. S3, Supplementary Material online). In total, we identified 2,341,224 SNPs in the 58 inferred haplotypes. Our empirically determined false discovery rate indicated a low mean false positive (FP) and false negative (FN) rate (2.6383e-04 and 1.6531e-08, respectively; supplementary fig. S4, Supplementary Material online).

#### Buildup of LD during Experimental Evolution

With a census size of 1,000 individuals, the population size in our experimental population was considerably smaller than that of natural *D. melanogaster* populations (e.g., Karasov et al. 2010). This reduction in population size in combination with selection caused by adaptation to a new environment is expected to have a considerable impact on LD in our experimental populations. We investigated the change in LD on two different scales, short range (up to 1 kb), beyond which typically no LD is found in natural *D. melanogaster* populations, and long range (>1 kb) through full chromosome haplotype information and compared these with changes from neutral simulations.

##### Short Range LD.

**Fig. 1.**
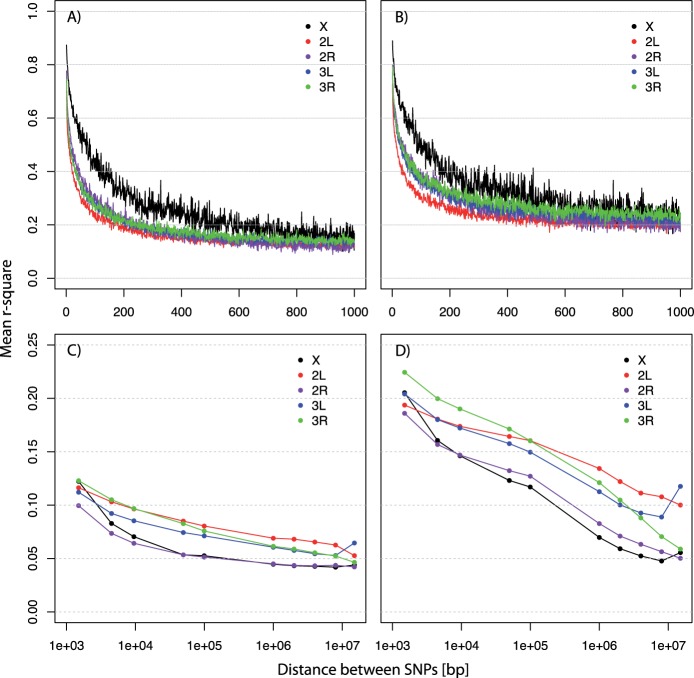
Decay of LD with physical distance for the major chromosome arms. Decay of short range LD in (*A*) the base and (*B*) the evolved population and of long range LD in (*C*) the base and (*D*) the evolved population. LD estimates (*r*^2^) are based on SNPs that are polymorphic in the base and the evolved population, with at least 24 haplotypes. Data points for long range LD estimates (dots) present averages of 1-kb windows for distances of 2, 5, 10, 50, 100 kb, 1, 2, 4, 8, and 15 Mb. LD decreases rapidly within 200 bp in both populations but is generally higher in the evolved population, where it also extends over longer distances.

As anticipated from previous studies (e.g., Langley et al. 2012; Mackay et al. 2012), short range LD in the base population was higher on the X chromosome compared with the autosomes and decreased rapidly with physical distance. Decay of LD was strongest within the first 200 bp and decreased to an *r*^2^ value slightly above 0.1 at 1-kb distance (fig. 1*A*). We note that this relatively high value is not a specific feature of our base population, but reflects the moderate sample size used to estimate *r*^2^ (Weir and Hill 1986). After 67 generations of experimental evolution in the hot laboratory environment, LD increased almost uniformly by Δ*r*^2^ of approximately 0.075–0.1 within distances up to 1 kb (fig. 1*B* and supplementary fig. S5*A*, Supplementary Material online). For SNPs separated between 0.9 and 1 kb, *r*^2^ was almost twice as high in the evolved population (base: *r*^2^ ∼ 0.13 vs. F67: *r*^2^ ∼ 0.22). Similar increases could also be reproduced from simulations under neutrality alone with *N*_e_ = 200 (for *N*_e_ estimates, see Orozco-terWengel et al. 2012; Tobler et al. 2014). Due to the reduction population size, LD in the simulations increased uniformly for distances up to 1 kb from *r*^2^ ∼ 0.13 in the base to *r*^2^ ∼ 0.26 in generation 67 (distances between 0.9 and 1 kb) (supplementary fig. S5*C*, Supplementary Material online). As expected under neutrality LD increased strongest on the X chromosome due to the lower effective population size (supplementary fig. S5*C*, Supplementary Material online). Interestingly, in the experimental data LD increase was strongest on the autosomes (supplementary fig. S5*A*, Supplementary Material online), suggesting a stronger impact of selection on the autosomes.

##### Long Range LD.

Our analysis further showed that, in the base population, long range LD continues to decrease with distance, leveling off at *r*^2^ ∼ 0.05 within 1–10 Mb (fig. 1*C*). This value is very close to the mean background *r*^2^ of approximately 0.04 determined from random SNP pairs between chromosomes (see Materials and Methods). As expected, in the evolved population mean LD was slightly higher and extended over larger distances. The decay of LD differed between chromosomes. Mean *r*^2^ estimates of approximately 0.05 similar to the base population were reached on X, 2R, and 3R within 10–15 Mb but were not for 2L and 3L (fig. 1*D* and supplementary fig. S5*B*, Supplementary Material online). Interestingly, we noted a similar increase in LD for tightly spaced SNPs and those separated up to 100 kb (supplementary fig. S5*B*, Supplementary Material online). This uniform shift in LD indicates that only a few recombination events have occurred during the experiment. Thus, a haplotype that increases in frequency typically stays intact for several kilobases and thereby generates the same increase in LD for closely and more distantly linked sites (see supplementary fig. S6, Supplementary Material online, for an alternative visualization confirming frequency change of large haplotype-blocks). These findings were also confirmed in the neutral simulations, where a small but clear increase in LD was present up to distances of 100 kb. In contrast to the experimental data, we did not see an increase in LD for distances ≥8 Mb in our simulations (supplementary fig. S5*D*, Supplementary Material online). This suggests that increases at such distances might require additional features such as large inversions or strong selection on rare haplotypes.

On average, LD increase in the simulated data was marginally higher (*r*^2^ ∼ 0.05) than in the experimental data in which selection was additionally operating (supplementary fig. S5, Supplementary Material online). This suggests that the effective population size in the experiment was probably higher than the simulated *N*_e_ of 200.

##### Dependence of LD Increase on Initial LD.

**Fig. 2.**
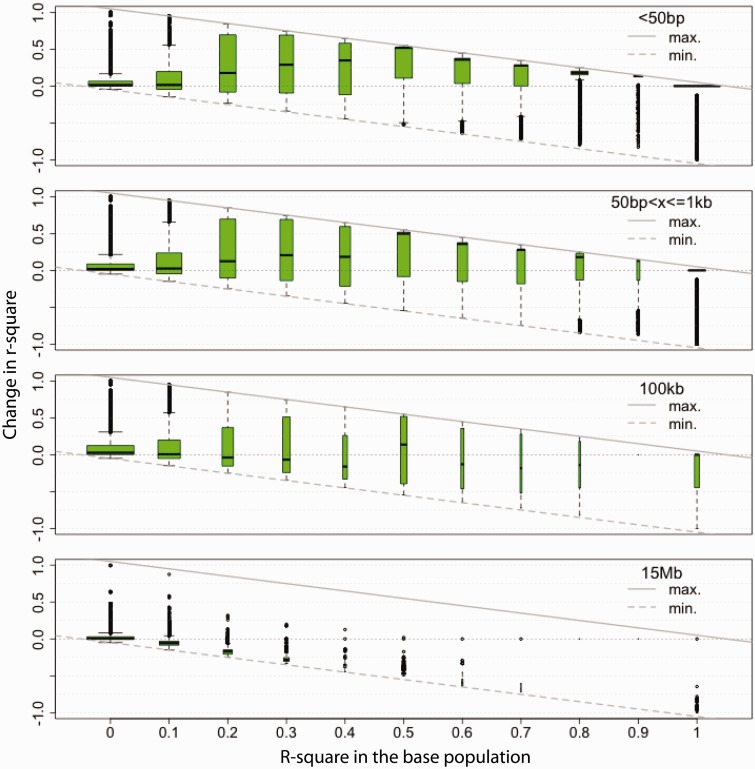
Change in LD after 67 generations of evolution depends on the initial LD levels. Results for chromosome arm 3R (plots for remaining chromosome arms are in supplementary fig. S7, Supplementary Material online). Boxplots indicate LD change after 67 generations (*y* axis) for different categories of initial LD values (*x* axis). In each row, SNPs with different spacing are shown: ≤50 bp (first row), 51 bp–1 kb (second row), 99–100 kb (third row), and 14.999–15 Mb (fourth row). Solid and dashed diagonal lines indicate the maximum and minimum possible change in LD, respectively. Width of the boxes is scaled by category size (i.e., number of pairwise comparisons). *r*^2^ was calculated for matched SNPs polymorphic in both populations. Across all chromosome arms the following patterns are present: 1) In the short range (≤1 kb), where recombination events are very rare, LD increase is more likely if initial LD is present; 2) if new recombination events are more frequent (distances ∼100 kb), LD increase is no longer facilitated by initial LD; LD in the starting population typically decreases for large distances when new recombination events are frequent (∼15 Mb); 3) for the majority of SNPs, *r*^2^ takes extreme values (close to either zero or one); and 4) over short distances most SNPs experience change in LD as initial LD is high and recombination during the experiment can be neglected.

In addition to the general pattern of LD increase during experimental evolution, we investigated LD dynamics in relation to LD in the starting population. LD is affected by two opposing forces: 1) Frequency changes through genetic drift and/or selection and 2) recombination. The presence of LD implies that at least one haplotype occurs at a lower frequency than expected under linkage equilibrium. This inequality increases the probability of losing the least frequent haplotype(s) through random sampling. Hence, in the absence of recombination a positive correlation between initial LD and LD increase is expected. Consistent with this idea, we observed that closely linked SNPs with higher initial LD showed a larger increase in LD (SNPs separated by <=1 kb; fig. 2 and supplementary fig. S7, Supplementary Material online). Although high LD SNPs (>0.7) were limited in their maximal increase, intermediate LD SNPs (0.3–0.7) had a median LD increase of almost 0.5. The correlation between initial LD and LD increase was weaker for SNPs at greater distances and higher recombination probability, which also had fewer SNPs with high initial LD (fig. 2). For widely spaced SNPs (∼15 Mb), new recombination events were frequent enough that SNPs with high LD in the base population almost always became uncoupled (fig. 2 and supplementary fig. S7, Supplementary Material online). As expected, these general patterns were also present in the simulated data (data not shown).

A closer inspection of different long range LD classes indicated that the increase seen in mean LD was mainly due to SNPs with initial LD close to zero, which reached very high LD in the evolved population (supplementary fig. S8, Supplementary Material online). This suggests that long range LD increased due to only a few haplotypes, which might at least temporarily have escaped recombination.

##### Influence of Inversions and Low Recombination Regions.

**Fig. 3.**
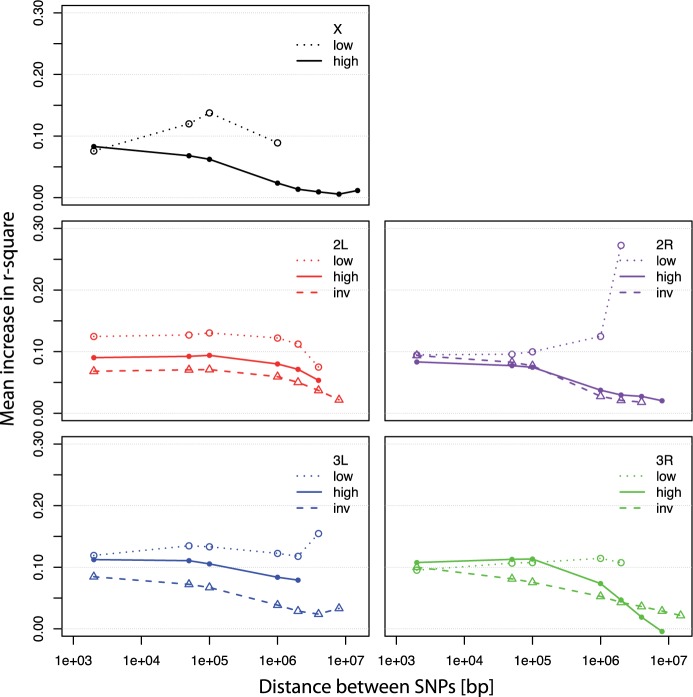
Increase in long range LD within 67 generations for three different categories of genomic regions: 1) Low recombining regions (dotted lines and open circles), 2) genomic regions covered by cosmopolitan inversions segregating in our populations (table 1) (dashed lines and open triangles), and 3) the remaining high recombining regions (solid lines and full circles). Borders between the different categories are provided in supplementary table S3, Supplementary Material online. LD estimates (*r*^2^) are based on SNPs that are polymorphic in the base and the evolved population, with a minimum of 24 haplotypes. Data points for long range LD estimates (circles, triangles) present averages of 1-kb windows for distances of 2, 50, 100 kb, 1, 2, 4, 8, and 15 Mb. Contrary to expectations inversion regions show reduced or similar amount of LD increase in comparison to high recombining regions, whereas low recombining regions often show higher LD increase.

One possible explanation for the increase of long range LD in the evolved populations is the presence of inversions, which suppress recombination and often harbor higher levels of LD than noninverted types (Corbett-Detig and Hartl 2012; Kapun et al. 2013). Six cosmopolitan *D. melanogaster* inversions (*In(2L)t*, *In(2R)Ns*, *In(3L)P*, *In(3R)Payne*, *In(3R)C*, and *In(3R)Mo*) are segregating in our experimental populations (Kapun et al. 2013). Based on inversion-specific SNPs, we inferred initial inversion frequencies from the haplotype data. They ranged from 0.03 to 0.34 with all inversions showing a reduction or no change in frequency in the evolved haplotypes (table 1). Surprisingly, we did not find evidence for a stronger LD increase for genomic regions spanned by inversions. Rather, they showed similar or even smaller LD increases compared with the high recombining regions (fig. 3). This could have multiple causes: 1) In the base population, inverted and noninverted regions have similar LD (supplementary fig. S9, Supplementary Material online); 2) due to low inversion frequencies (apart from *In(2L)t* at 0.34), only a small fraction of the individuals were heterozygous for the inversion and thus had restricted recombination; and 3) none of the inversions appeared to be under positive selection apart from *In(3R)C* (see also Kapun et al. 2013 for changes in inversion frequency over time). We note that the limited influence of inversions on the increase in LD may be specific to our study and other experiments with different inversion frequencies and selection pattern may find a more pronounced effect of inversions on the increase in LD.

**Table 1.** Inversions in the Sequenced Haplotype Data.

	29 Base Haplotypes (F0)			29 Evolved Haplotypes (F67)			
	Inversion Carriers	# Inversion Carriers	Inversion Frequency	Inversion Carriers	# Inversion Carriers	Inversion Frequency	Change in Frequency
*In(2L)t*	1, 6, 7, 10, 12, 13, 19, 22, 28, 29	10	0.34	2, 5, 7, 14, 15, 17, 22, 24, 25	9	0.31	−0.03
*In(2R)Ns*	6	1	0.03	0	0	0.00	−0.03
*In(3L)P*	2, 7, 22, 23, 28	5	0.17	2	1	0.03	−0.14
*In(3R)K*	None	0	0.00	None	0	0.00	0.00
*In(3R)C*	19, 20, 22, 23, 26, 28	6	0.21	2, 14, 25, 27, 28	5	0.17	−0.03
*In(3R)Mo*	1	1	0.03	15	1	0.03	0.00
*In(3R)Payne*	8, 11, 16, 21, 24, 25	6	0.21	0	0	0.00	−0.21

Although genomic regions with segregating inversions were not a good predictor of higher LD increase, we found that LD increase was typically higher in low recombination regions relative to high recombination regions (fig. 3 and supplementary fig. S9, Supplementary Material online). Nevertheless, as long range LD also increases in high recombination regions (fig. 3), other factors besides low recombination have contributed to the observed increase in long range LD in our experimental populations.

### Inference of Selected Haplotype-Blocks during Experimental Evolution

Our second analysis combines data of the 29 sequenced base haplotypes with Pool-Seq time series data to infer blocks that increase in frequency during experimental evolution. Haplotype-blocks were identified through the rise of marker SNPs of the base haplotypes across replicates in a sliding window. The integrity of haplotype-blocks during the experiment was verified through the 29 evolved haplotypes from generation 67.

#### Haplotype-Blocks Rising from a Low Frequency

**Fig. 4.**
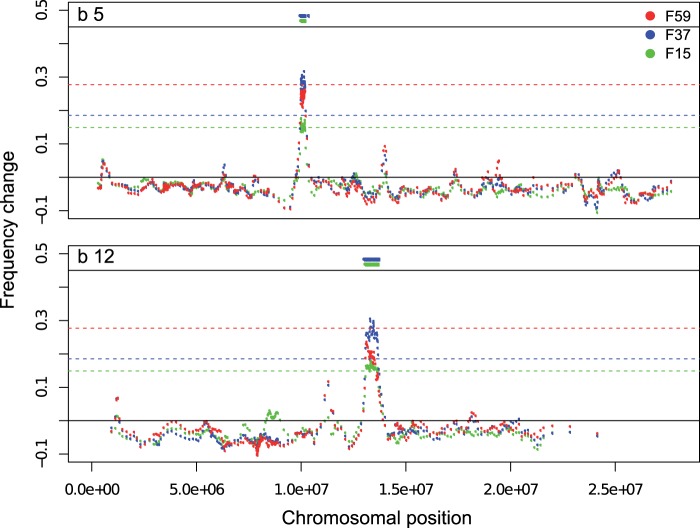
Identification of two different haplotype-blocks (b5, b12) on 3R. Singletons specific to either haplotype, b5 (upper panel), or b12 (lower panel), were identified from 29 haplotypes. In a sliding-window analysis, frequency changes of singleton-windows (inferred from Pool-Seq) are plotted for three different comparisons of the base to generation F15, F37, and F59. Dashed lines present thresholds for haplotype-block identification for the respective generations based on mean frequency changes of the top 2,000 CMH candidates (see Materials and Methods). Short solid lines above indicate areas of identified haplotype-blocks.

**Fig. 5.**
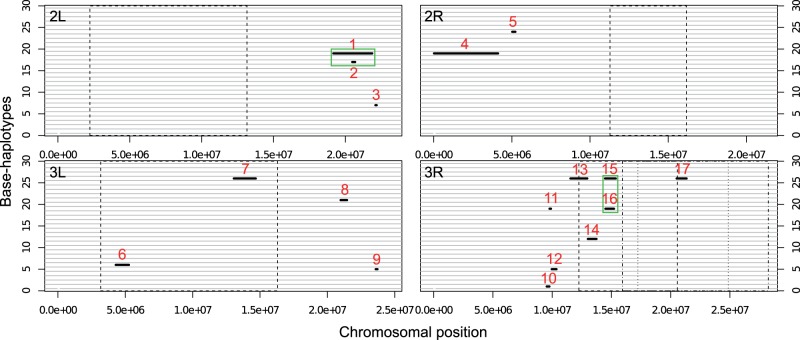
Overview of all 17 haplotype-blocks in the base population. Each horizontal line represents one of the 29 sequenced haplotypes in the base population (b1–b29). Each panel represents an autosomal chromosome arm (no haplotype-block was detected on the X-chromosome). The haplotype-blocks are numbered from 1 to 17 and their position is indicated by a bold line. A visual explanation of how haplotype-blocks were identified is given in figure 4 and supplementary figure S11, Supplementary Material online. Solid rectangles highlight haplotype-blocks that overlap the same genomic region (i.e., 1 & 2 and 15 & 16). The dashed lines indicate chromosomal positions of the cosmopolitan *D. melanogaster* inversions *In(2L)t*, *In(2R)Ns*, *In(3L)P*, *In(3R)Payne*, *In(3R)C*, *In(3R)Mo* (Ashburner and Lemeunier 1976; Corbett-Detig and Hartl 2012).

Tobler et al. (2014) recently proposed an alternative explanation for the increase in long range LD in experimentally evolving populations. Strong selection on rare haplotypes could create pronounced long range LD as the increase in frequency of a rare haplotype increases stochastic loss of alleles on all other haplotypes. Using Pool-Seq data, Orozco-terWengel et al. (2012) and Tobler et al. (2014) described a region of approximately 1.5 Mb, in which many, rare alleles increased in frequency by about 27% over 15 generations. Indeed, we mapped many of the candidate SNPs in this region to a single haplotype in the base population (supplementary fig. S10, Supplementary Material online). Reasoning that this may be a more general phenomenon, which could explain the long range LD in our data, we systematically searched for haplotype-blocks in the base population that increased to high frequency during the experiment. In a sliding window analysis, we identified haplotype-blocks in the base population which showed a strong frequency increase of haplotype-specific SNPs (i.e., those SNPs that were singletons in the 29 haplotype sequences of the starting population; see Materials and Methods). Only windows containing singletons, for which the frequency increase was greater than a time point specific threshold (0.1489 [F15], 0.1851 [F37], or 0.2771 [F59]), were included in such a haplotype-block (fig. 4 and supplementary fig. S11, Supplementary Material online; see Materials and Methods). After combining windows within 2-cM distance, we obtained 17 haplotype-blocks. The blocks originated from ten different base haplotypes on chromosomes 2 and 3, and covered approximately 13% of the analyzed genome (fig. 5, table 2). In two cases, two haplotype-blocks overlapped in the same genomic region (fig. 5). The length of the haplotype-blocks ranged from 0.082 to 4.095 Mb, corresponding to a genetic distance between 0.005 and 4.84 cM (table 2).

**Table 2.** Features of the 17 Haplotype-Blocks.

Haplotype- Block	Haplotype	Chr.	Start Position	End Position	Length (bp)	Local Recomb. Rate (cM/Mb)	Genetic Linkage (cM)^a^	# Putative Candidate Genes^b^	Time Points Supported By^c^
1	b19	2L	19164025	21867353	2,703,329	0.36	0.97	180	F15, F37
2	b17	2L	20478452	20677572	199,121	0.43	0.09	15	F59
3	b7	2L	22094360	22176712	82,353	0.06	0.005	10	F59
4	b19	2R	22338	4116872	4,094,535	0.50	2.05	186	F15, F37
5	b24	2R	5022544	5224847	202,304	1.34	0.27	25	F37
6	b6	3L	4292577	5266513	973,937	3.44	3.36	48	F37
7	b26	3L	13054620	14681637	1,627,018	1.46	2.38	94	F37, F59
8	b21	3L	20981296	21454727	473,432	0.13	0.06	35	F37
9	b5	3L	23586018	23715391	129,374	0.05	0.01	1	F15
10	b1	3R	9540775	9768106	227,332	1.92	0.44	20	F15, F37
11	b19	3R	9780631	9894750	11,412	5.28	0.60	NA^d^	F37
12	b5	3R	9962187	10363636	40,145	3.37	1.35	35	F15, F37
13	b26	3R	11544110	12961670	1,417,561	1.47	2.08	107	F15, F37
14	b12	3R	13013645	13739370	725,726	1.62	1.18	44	F15, F37
15	b26	3R	14462622	15368971	90,635	5.32	4.84	66	F15, F37
16	b19	3R	14498346	15216055	71,771	5.62	4.05	45	F15, F59
17	b26	3R	20506391	21340829	834,439	2.52	2.10	79	F37

^a^The local recombination rate was estimated by averaging the recombination rates for all 100-kb windows experimentally determined by Comeron et al. (2012) within the respective haplotype-block.

^b^Putative candidate genes are located in the haplotype-block region and contain at least one SNP frequency less than 0.1 in the base population of the haplotype-block allele.

^c^“Time points supported by” indicates due to which generations the haplotype-block was identified, that is, exceeded the outlier threshold (see Materials and Methods).

^d^Candidate genes were not determined because base-haplotype b19 was identified to be a similar but different to the haplotype rising in the experiment (supplementaryfigs. S12B and S13, Supplementary Material online).

Haplotype-blocks were not preferentially associated with common *D. melanogaster* inversions. Out of seven haplotype-blocks falling into genomic region covered by at least one inversion, only one, haplotype-block 17, was located in an inversion (fig. 5, tables 1 and 2).

#### Selection Changes Haplotype-Block Frequencies

**Fig. 6.**
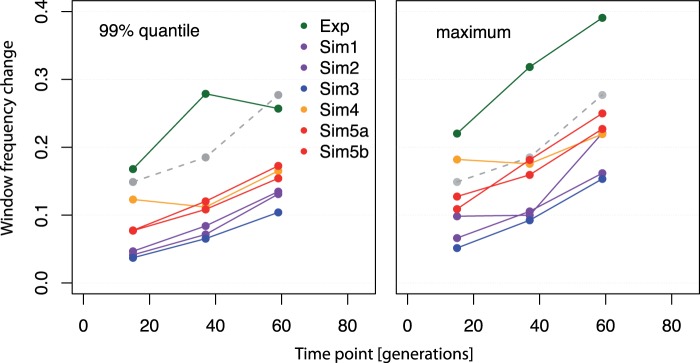
Frequency change of singleton-SNP windows in experimental and simulated data. Dots show the frequency changes from the base to respective generation (F15, F37, and F59) of the singleton-SNP windows used for haplotype-block identification (see Materials and Methods). The left panel shows the 99% quantile and right panel the maximum frequency change of all singleton-SNP windows. Different colors show the experimental data (Exp) and the neutral simulations (Sim1–3: *N*_e_ = 250, Sim4–5: *N*_e_ = 150, see Materials and Methods, supplementary table S1, Supplementary Material online). The gray dots joined by the dashed line represent the thresholds used for haplotype-block identification.

Our procedure for haplotype-block identification relied on empirical cutoffs obtained from the 2,000 most significant SNPs in pairwise comparisons between the base and the corresponding generation. Although this procedure already suggests that haplotype-blocks are not neutrally evolving (Orozco-terWengel et al. 2012; Tobler et al. 2014), we performed computer simulations to confirm the suggested departure from neutrality. Whole-genome forward simulations (see Materials and Methods) based on conservative population size estimates (Orozco-terWengel et al. 2012; Tobler et al. 2014) resulted in smaller frequency changes of singletons than observed in the experimental data (fig. 6 and supplementary table S1, Supplementary Material online). Only in one out of five simulations we identified a single putatively selected haplotype-block (length 22.89 kb). As the experimental evolution study resulted in 85-times more haplotype-blocks than neutral computer simulations, we conclude that selection caused the increase in frequency of haplotype-blocks.

#### Are Haplotype-Blocks Recombining during the Experiment?

**Fig. 7.**
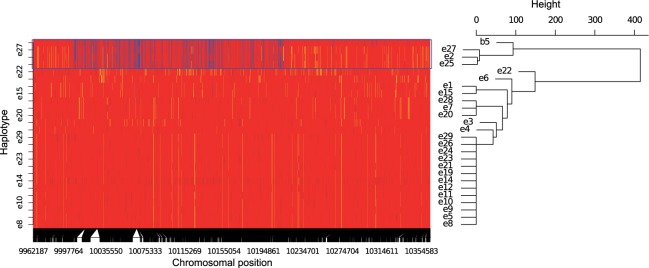
Haplotype-block 12 and the evolved haplotypes from generation 67. Haplotype-block 12 from the base population is labeled with b5; 25 evolved haplotypes are labeled with e (four evolved haplotypes were excluded as <20% of their bases in that region were called). The left panel represents the haplotype block structure. Each horizontal line corresponds to one haplotype and each column to a singleton-SNP position within the base haplotypes. SNPs specific to haplotype-block 12 are indicated in blue, yellow indicates the remaining singletons in the base population. The major allele in the base population is shown in red. Missing data are shown in gray. The right panel shows a cladogram (average linkage clustering) based on singleton-SNP sharing. The blue rectangle marks the evolved haplotypes that cluster with b5. The core of the haplotype-block is shared among the three evolved haplotypes indicating no recombination in this region. As toward the end of the blocks singleton markers for b5 are scarce, it might be that the block has been called slightly too long.

An important question is whether, and to what extent, the haplotype-blocks were broken up by recombination during the 67 generations of evolution in the hot laboratory environment. We identified recombination events by comparing the haplotype-block sequence from the base to the haplotypes in generation F67. A visual inspection of the singletons specific to the haplotype-block in the base suggested a low, but variable degree of recombination among the evolved haplotype-blocks (fig. 7 and supplementary fig. S12, Supplementary Material online). To quantify the similarity of the evolved haplotype-blocks with the ancestral one, we calculated pairwise distances based on allele sharing using all SNPs. If the haplotype-block structure is fully preserved, the distance to evolved haplotypes should be close to zero. On the other hand, if recombination has disrupted the block, the inferred distance is proportional to the exchange of genetic material. We detected a variable degree of haplotype-block persistence (fig. 7 and supplementary figs. S12 and S13, Supplementary Material online). The evolved haplotypes for haplotype-blocks 1, 2, 8, and 15 showed no evidence of recombination, whereas for haplotype-blocks 3, 4, 5, 6, 10, 12, 13, 16, and 17 a small and for blocks 7 and 14 a slightly higher amount of recombination at block ends could be detected. Haplotype-block 9 was not similar to any derived haplotypes of time point F67 (replicate R2) (supplementary figs. S12*A* and S13, Supplementary Material online). Though haplotype-block 11 shared several marker SNPs with the haplotype-block that increased in frequency during the experiment, several markers also clearly indicated that the increasing haplotype was not identical to the corresponding base-haplotype (supplementary figs. S12*B* and S13, Supplementary Material online). We conclude that our definition of haplotype-blocks is justified as we identified only small amounts of recombination during 67 generations in our experiment.

#### Enrichment of Candidate SNPs in Haplotype-Blocks

**Fig. 8.**
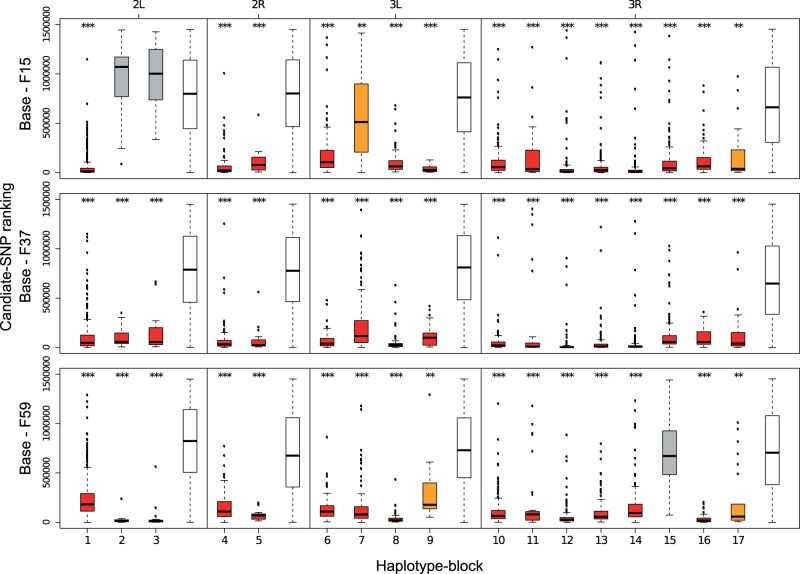
Enrichment of candidate SNPs in haplotype-blocks. To test whether haplotype-block-specific SNPs are among the most significant SNPs in the Pool-Seq data, we plot their rank among all SNPs tested with the Pool-Seq data. Three comparisons are shown: base–F15, base–F37, and base–F59, respectively. For comparison, the rank of all singletons on the same chromosome arm that are not part of a haplotype-block is shown as white boxplots. Boxplots for haplotype-block singletons are shaded according to their differentiation to the nonhaplotype-block background for the respective chromosome arm (Mann–Whitney *U* test): ***FDR < 10^−10^, **FDR < 10^−3^, or no significant differentiation. Singletons in all 17 haplotype-blocks show candidate enrichment for at least one time point. Enrichment over time differs between the blocks.

Haplotype-blocks move as a unit during the experiment and show signatures of selection. We expected that singletons in haplotype-blocks are overrepresented among candidate SNPs from Pool-Seq analyses as they exhibit the most pronounced frequency changes in a selected haplotype-block. Indeed, we find the expected enrichment of candidates when we rank haplotype-block-specific SNPs relative to all other singletons based on the significance of the Cochran–Mantel–Haenszel (CMH)-test in three different comparisons: Base-F15, base-F37, and base-F59 (fig. 8). Nevertheless, the pattern of enrichment differs among haplotype-blocks. Several haplotype-blocks show a similar enrichment for all time points (blocks 1, 4, 5, 6, 8, 10–14, and 16), whereas others are either only enriched at the beginning (block 9) or the end (block 2, 3, and 7) of the experiment (fig. 8). Four haplotype-blocks (2, 3, 7, and 16) exhibit an increase in candidate enrichment over time, whereas five haplotype-blocks (1, 4, 9, 14, and 15) show a decrease (Linear regression: rank ∼ time point, FDR < 0.001; supplementary fig. S14, Supplementary Material online). Overall, we find an enrichment of candidate SNPs in the genomic regions covered by the haplotype-blocks. Although blocks only covered approximately 13% of the genome, between 42% (base-F15) and 46% (base-F59) of the candidate SNPs mapped to block regions.

#### Competing Haplotype-Blocks

In two genomic regions, we identified more than one haplotype-block (fig. 5). On chromosome 2L one haplotype-block is nested within the other, whereas on chromosome 3R the competing haplotype-blocks are of similar size. Interestingly, the haplotype-blocks covering the same genomic region have different dynamics (supplementary fig. S15, Supplementary Material online). Haplotype-block 1 initially rises quickly and plateaus at a frequency between approximately 0.2 and 0.3 in all replicates, whereas the frequency of its counterpart increases later but continuously. Similarly, haplotype-block 15 is the first one to increase in frequency, but once haplotype-block 16 becomes more abundant, the frequency of haplotype-block 15 decreases slightly (supplementary fig. S15, Supplementary Material online). Note that replicate R2 had a different dynamics for haplotype-block 16 (supplementary fig. S15, Supplementary Material online).

#### Temporal Dynamics of Haplotype-Blocks

Previously, analyses of the same experiment reported the allele frequency trajectories of the top 2,000 candidate SNPs for the first two time points, generation 15 and 37 (Orozco-terWengel et al. 2012). The authors made two important observations. First, candidate gene sets were largely nonoverlapping between subsequent time points. Second, frequencies of the top candidate alleles from generation 15 rose quickly and subsequently plateaued, whereas candidates from generation 37 increased continuously. Several hypotheses were put forward to explain the plateauing of candidate SNPs at subsequent time points, including marginal overdominance (see Sellis et al. 2011), polygenic adaptation (see Chevin and Hospital 2008), and antagonistic pleiotropy (Orozco-terWengel et al. 2012). However, the plateauing may also be due to purely technical reasons similar to the phenomenon of the “winners curse” (Burke and Long 2012). In this case, it is likely that, for some loci, which were identified in a comparison between the starting population and generation 15, the allele frequency difference will be overestimated, resulting in a slight drop or plateau in the subsequent time point. Although Orozco-terWengel et al. (2012) made some effort to rule out this explanation, it remains a valid concern in pairwise comparisons that perform a large number of tests. Through the combined use of haplotypes and Pool-Seq data, we have identified putatively selected haplotype-blocks that were enriched for candidate SNPs at several time points during experimental evolution. The rapid increase of these large haplotype-blocks during our experiment provides other possible explanations for the previously reported plateauing of candidate loci. First, several candidates may plateau because they are neutral hitchhikers that cease to increase in frequency once they have been uncoupled by recombination. Second, as the haplotype-blocks are large, it may be that some blocks contain recessive deleterious allele(s) that become visible to selection in homozygotes upon obtaining higher frequencies. Third, large selected haplotype-blocks might temporarily interfere with each other because they overlap in the same genomic region, a phenomenon called the Hill–Robertson effect (Hill and Robertson 1966). More detailed analyses of the selected alleles are needed to distinguish between the different hypotheses for the observed plateauing.

#### Putative Candidate Genes in Rising Haplotype-Blocks

The large frequency increase of the haplotype-blocks suggests the presence of at least one favorable variant in each haplotype-block. As each haplotype-block started from a low frequency in the base population, we reasoned that causative alleles were initially also at low frequencies. Otherwise, the increase of the beneficial allele would have been distributed over several haplotypes and such a strong increase of a single haplotype would not have been observed. Thus, we identified putative candidate genes as those containing at least one low frequency allele (<0.1) in the base population within a haplotype-block. Most of the haplotype-blocks harbored a large number of putative candidate genes (at least 10; table 2 and supplementary table S2, Supplementary Material online), precluding the identification of the target of selection. However, there was only a single candidate gene on haplotype-block 9, Argonaute 3, which was supported by 42 intronic and 3 intergenic (downstream) candidate SNPs (supplementary table S2, Supplementary Material online). Argonaute 3 is a component of the “ping-pong” model of transposable element (TE) silencing in the *Drosophila* germ line (Brennecke et al. 2007; Obbard et al. 2009). Because (temperature) stress can induce TE activity (Capy et al. 2000) and most TE insertions are deleterious, we speculate that the selected Argonaute 3 variant may counterbalance the temperature effect on TE activity in the evolving *D. melanogaster* population.

#### Implications from Haplotype Analysis for Design and Analysis of E&R Studies

Here, we demonstrated that in experimental evolution studies haplotype-blocks ranging from several kilobases up to few megabases could strongly increase in frequency within a few generations. As these haplotype blocks harbor many private SNPs, this increase results in a large number of false candidates, which are indistinguishable from the causative variant(s).

On the one hand this result suggests that haplotype information, in particular of the experimental starting population, along with temporal frequency data will be important to localize independent targets of selection. In this study, we localized a few tens of selected haplotype-blocks starting from low frequencies in the base population with only 18–20% of the base-haplotypes being characterized. It is apparent that additional targets of selection and an increased accuracy of haplotype frequencies estimates can be obtained when more founder haplotypes are known (see Materials and Methods). One possibility to obtain more detailed information about founder haplotypes is the use of sequenced natural isolates, such as the DGRP lines (*Drosophila* Genetic Reference Panel, Mackay et al. 2012) or recombinant inbred lines obtained from a few founder genotypes (e.g., *D. melanogaster* in King et al. 2012; *Saccharomyces cerevisiae* in Cubillos et al. 2013).

On the other hand, computer simulation studies suggest the power to detect causative variants increases with number of haplotypes in the starting populations (Kofler and Schlötterer 2013; Baldwin-Brown et al. 2014). As the number of lines/strains with a known genomic sequence are typically lower than a powerful experimental design would require, we think that future E&R studies are better advised to maximize the number of founder haplotypes and replicates. Both factors will reduce the influence of founder haplotype blocks: A larger number of starting haplotypes increases the opportunity for recombination before the start of the experiment, that is, the beneficial mutation will more often be located on multiple haplotypes. More replicates will increase the opportunity for recombination during the experiment, which reduces the size of the haplotype-block that is consistently associated with the beneficial mutation(s).

## Conclusions

The accuracy to localize genomic targets of selection in experimental evolution studies depends on LD in the starting population and the number of recombination events during the experiment. Analyzing full chromosomal haplotypes in combination with time series data, we demonstrated that for commonly used experimental conditions new recombination events are relatively infrequent. This suggests that LD in the starting population is the key determinant for the mapping resolution of E&R experiments for commonly used experimental parameters. In our experiment, we identified coarse mosaics of the starting haplotypes and an almost uniform increase in LD up to distances of a few hundred kilobases. Thus, favorable low frequency alleles occurring only on rare haplotypes will result in long range hitchhiking due to the frequency increase of the entire haplotype-block. Importantly, all singletons private to the selected haplotype will bear the signature of selection, resulting in a large number of FPs.

The key for the success of future E&R studies to map causal variants depends on an experimental design that allows uncoupling of selected and neutral variants (see also Kofler and Schlötterer 2013; Baldwin-Brown et al. 2014). Although we did not find strong evidence that segregating inversions increased LD in the comparison of two time points for a single replicate, inversions are still likely to affect the mapping resolution on E&R studies because they 1) increase LD in the base population (Corbett-Detig and Hartl 2012; Kapun et al. 2013) and 2) reduce recombination in heterozygotes. Consequently, if variants associated with an inversion were selected, they could result in many FPs and we therefore recommend the use of species with few segregating inversions such as *D. simulans*. As hitchhiking is pervasive when strong selection acts on an allele private to a single haplotype it should be aimed to avoid experimental designs, in which a single haplotype sequence can experience a strong and fast frequency increase. If multiple copies of identical haplotypes are used in the founder population, this could facilitate the fast increase of a single selected haplotype sequence. Moreover, increasing the experimental population size, number of replicates, and the number of founder chromosomes (see also Kofler and Schlötterer 2013) in combination with the use of different haplotypes in replicated starting populations should similarly reduce the chance of a rapid increase of a kilobase- to megabase-sized haplotype sequence and thereby improve the mapping resolution.

Although selected haplotype-blocks impede the mapping of the favorable mutation, they could also be beneficial for studying the trajectories of selected alleles. As many haplotype-block-specific SNPs can be studied, it is possible to follow the allele frequency of selected blocks throughout the experiment at a higher precision, and with more confidence, than is possible for single SNPs.

## Materials and Methods

### Experimental Populations and Selection Regime

This report is part of an ongoing experimental evolution study (see Orozco-terWengel et al. 2012; Kapun et al. 2013; Tobler et al. 2014; Versace et al. 2014). Briefly, *D. melanogaster* from a natural Northern Portuguese population was caught in summer 2008 and established in the laboratory as 113 isofemale lines for five generations. Each base population was generated from five nonvirgin females of each isofemale line (=565 females), hereafter referred to as generation F0. The populations were kept as independent replicates in discrete generations at a census population size of approximately 1,000 individuals and a balanced sex ratio. The new temperature regime cycled every 12 h between 18 and 28°C coinciding with a dark and light period, respectively.

### Experimental Haplotype Inference

We determined full chromosomal haplotypes for 29 flies from each, the base population (b1–b29) and from an evolved population at generation F67 (replicate R2; e1–e29). One male from each of 29 isofemale lines (base population) or 29 males from replicate R2 in generation F67 were individually crossed to a virgin female from the inbred reference strain (y[1]; cn[1] bw[1] sp[1]) (supplementary fig. S1, Supplementary Material online). One female F1 offspring of each cross was used for DNA extraction and sequencing.

Sequencing of all 58 F1 individuals was performed with 100-bp paired-end reads on an Illumina HiSeq 2000. For each sample, DNA was extracted following a high salt extraction protocol (Miller et al. 1988) and fragmented using either NEBNext dsDNA Fragmentase (New England Biolabs, Ipswich, MA) or a Covaris S2 device (Covaris, Inc., Woburn, MA).

Libraries were prepared at different time points using protocols from various suppliers (details are available on request). To account for the possibility of residual heterozygosity in our reference strain, we used a sequenced pool of ten females of our reference strain (PRJEB4952: SAMEA2247828) (Kapun et al. 2013) and accounted for segregating variation in the subsequent analysis pipeline.

Preprocessing and mapping of reads were done using a standard pipeline described in Kofler, Orozco-terWengel, et al. (2011) and Kofler, Pandey, et al. (2011). Briefly, raw reads were trimmed at the 3′-end to remove low quality bases with trim-fastq.pl (parameters: –quality-threshold 20 –min-length 50). Reads were mapped with bwa (version: 0.5.8; parameters: -o 1 -n 0.01 -l 200 -e 12 -d 12) (Li and Durbin 2009) on a Hadoop Cluster with Distmap v1.0 (Pandey and Schlötterer 2013) to the reference genome of *D. melanogaster* (version 5.18). Libraries were demultiplexed and sorted prior to duplicate removal with Picard (version: 1.56; http://broadinstitute.github.io/picard/, last accessed November 27, 2014). Mapped reads were filtered for low quality with SAMtools (version: 0.1.9, parameters: -q 20 -f 0x0002 -F 0x0004 -F 0x0008) (Li et al. 2009). Quality filtered bam files of the different samples were converted into the mpileup format with SAMtools and subsequently to a synchronized pileup with Popoolation2 scripts (mpileup2sync.jar; parameter: –min-qual 20) (Kofler, Pandey, et al. 2011). Repeat sequences identified with RepeatMasker 3.2.9 (www.repeatmasker.org, last accessed Nov 27, 2014) together with 5-bp windows around indels were excluded from the synchronized pileup (identify-indel-regions.pl; parameter: –min-count 90 for a total of ∼17 HiSeq lanes) (Kofler, Pandey, et al. 2011).

Haplotypes were called from F1 individuals as described in Kapun et al. (2013) (supplementary fig. S1, Supplementary Material online). Briefly, paternal alleles were identified through the comparison of the F1 genotype to the reference strain. An alternate paternal allele (with respect to the reference allele) was called when its frequency was within the 90% binomial confidence interval for an expectation of 0.5. Haplotype base calls were performed for positions that were monomorphic in the resequenced reference and had a coverage greater than 9 and less than the maximum 2% coverage of the respective library. SNPs were called among all 58 haplotypes.

We determined an empirical false discovery rate for chromosomal regions with a low sequencing coverage. Assuming that a higher coverage is more likely to reflect the true SNP, we downsampled five highly covered libraries (>60-fold mean coverage; libraries b7, b11, b12, e12, and e16) to a coverage of 10. FP SNP calls (with respect to the reference strain) and FN SNP calls were estimated by comparing base calls between complete and downsampled libraries. Interestingly, these FP rates are very similar to the ones reported in Kapun et al. (2013), although an entirely different approach for false discovery rate estimation was used.

### Estimation of Short and Long Range LD from Haplotype Data

Linkage between SNP markers was estimated by *r*^2^ using custom scripts (supplementary material, script 1, Supplementary Material online). *r*^2^ values were calculated for all possible SNP-pairs for a given physical distance if the following conditions were met: 1) Both SNPs were polymorphic in the base as well as the evolved population and 2) the respective haplotype had a minimum coverage of 24. Calculation of LD between chromosomes was performed based on 2,000 SNPs randomly drawn from each chromosome. Mean *r*^2^ was determined for the base and the evolved population separately only using SNP-pairs for which the coverage was sufficient in both populations.

### Identification of Common *D. melanogaster* Inversions

Chromosomal inversions in the 58 haplotypes of the base and the evolved population were identified based on inversion-specific SNP markers (Kapun et al. 2013). For the inversions *In(2L)t*, *In(2R)Ns*, *In(3L)P*, *In(3R)Payne*, *In(3R)C,* and *In(3R)Mo,* the number of markers ranged between 22 and 362, which allowed for unambiguous identification of inversion haplotypes. The categorization of inversion and remaining low and high recombining regions is based on borders defined in supplementary table S3, Supplementary Material online.

### Temporal Allele Frequencies through Pool-Seq

Allele frequencies for different time points of the experiment were estimated from Pool-Seq data (Futschik and Schlötterer 2010). We used sequencing data for all three replicates from time points F0, F15 (F23 for replicate R2), F37 (Orozco-terWengel et al. 2012), and F59. Replicates for generation F59 in the hot environment were sequenced by 100-bp paired-end sequencing on an Illumina HiSeq 2000. For each replicate, 500 females were pooled for DNA extraction using a high salt extraction protocol (Miller et al. 1988). Genomic DNA was sheared using a Covaris S2 device (Covaris, Inc. Woburn, MA, USA), and paired-end libraries were prepared using the TruSeq v2 DNA Sample Prep Kit (Illumina, San Diego, CA). After ligation of barcoded adapters, libraries were purified on Qiagen columns (Qiagen, Hilden, Germany), pooled in approximately equal amounts, jointly size-selected for a mean insert size of 300 bp on agarose gels, and amplified with ten polymerase chain reaction cycles prior to sequencing.

Read processing, mapping, and SNP calling were identical to the pipeline described in Tobler et al. (2014). Following Tobler et al. (2014), ranks of candidate loci (loci of putatively selected alleles) were determined for all approximately 1.45 million SNPs identified in the Pool-Seq data between the base and the respective time point by the CMH test (Agresti 2002). The CMH test has been shown to outperform other test statistics in experimental evolution settings (Kofler and Schlötterer 2013).

### Identification of Haplotype-Blocks

We defined haplotype-blocks as continuous stretches of a haplotype in the base population that rise in the experiment more than expected under neutral drift. Haplotype-blocks were defined in the base population. First, we determined all singletons for each base-haplotype. Second, for each base-haplotype we used a sliding window of 20 singletons and an overlap of 15 singletons to characterize genomic regions in which a strong frequency increase could be detected in subsequent generations (haplotype-block). For each window, an analysis of variance (freq∼timepoint*repl) and posthoc Tukey test were performed estimating the frequency increase (as the lower boundary of a 95% confidence interval) between F0 and each of the remaining time points (F15, F37, and F59). Windows were defined as part of a haplotype-block if the estimated frequency increase for any of the three time points exceeded an empirical threshold. The threshold was determined as the minimum of the mean frequency changes across replicates of the top 2,000 ranked candidate loci for the respective time point. Multiple windows meeting the threshold criterion were combined to a single haplotype-block if they overlapped or the local genetic distance (Comeron et al. 2012) between two block windows was less than 2 cM. The haplotype-block identification was performed with custom scripts written in python and R (R Core Team 2013) (supplementary material, script 2, Supplementary Material online).

We estimate the frequency of the identified haplotype-blocks for different time points during the experimental evolution study by using singletons specific to the 29 known base-haplotypes. The accuracy of the approach depends on the percentage of known base-haplotypes, with an increasing fraction of the founder population being sequenced resulting in more accurate identification of haplotype-specific singletons. We evaluated the accuracy in our experiment, as well as an expected improvement in accuracy through the usage of larger known fractions, using genomic sequences of 158 DGRP lines (Mackay et al. 2012). We determine haplotype markers for different sets of known base-haplotypes and estimate haplotype frequencies for populations simulated under neutrality. Simulations were performed with MimicrEE (Kofler and Schlötterer 2013) using *N*_e_ = 200 without recombination from a starting population with a similar representation of all 158 DRGP base haplotypes for chromosomes X, 2L, and 3L. The frequency of each haplotype was estimated by the median marker frequency in sliding, nonoverlapping windows of 20 marker SNPs along the chromosome. As expected, we confirmed that the accuracy of the haplotype frequency estimate depends on the number of sequenced base-haplotypes. We noted, however, that already for 29 sequenced haplotypes (18% of the total number) provided robust haplotype frequency estimates with a mean deviation of approximately 0.01. Accuracy further increased with a mean deviation of approximately 0.0075, 0.0025 and 0.001 for 20%, 50% and 75% of known base-haplotypes (supplementary fig. S16 and table S4, Supplementary Material online), respectively.

### Neutral Simulations Including Haplotype Information

Neutral simulations were carried out 1) to exclude the possibility that observed haplotype-blocks could be explained by neutral genetic drift alone and 2) to obtain expectations of LD increase through the reduction in the census population size. We performed forward Wright–Fisher simulations including whole-genome haplotype information with MimicrEE (Kofler and Schlötterer 2013) using two different settings: 1) To rule out that haplotype-block could have occurred through drift alone five simulation runs were performed, each with three replicate populations for which the genomic haplotypes were recorded (at F0, F15 [F23 for R2], F37, and F59). Simulations differed by their haplotype sequences in the base population and the effective population sizes (*N*_e_) (supplementary table S1, Supplementary Material online). The base population was created from 158 haplotypes from either the DGRP lines (Mackay et al. 2012) or a population simulated under a neutral coalescent model (Bastide et al. 2013). 2) For estimating changes in LD, simulations were performed for one replicate recording haplotype data for the base population created from 158 haplotypes from the DGRP lines (Mackay et al. 2012) and generation 67. As in all simulations the targeted population size (2*N*_e_ haploid chromosome sets) was larger than 158 haplotypes, each haplotype was included multiple times, a procedure mimicking the setup of the experimental *D. melanogaster* populations. The simulation parameters were chosen to match the previously determined lower boundaries for the effective population size (Orozco-terWengel et al. 2012; Tobler et al. 2014). All simulations used the experimentally determined recombination rates by Comeron et al. (2012) translated with the recombination rate calculator by Fiston-Lavier et al. (2010).

Haplotype-block identification from neutral simulations matched the procedure for the experimental data. For each simulation, singleton-SNPs were called from 29 haplotypes in the base population (for simulation Sim5 haplotype-blocks were called for two different sets of 29 randomly chosen base-haplotypes, Sim5a/b, to test for variation due to haplotype sampling). To match the SNP calling procedure in the experimental data, we matched the coverage for each position to the real data by binomial sampling (details in Tobler et al. 2014). The allele frequency spectrum between experimental and simulated data matched very closely, demonstrating the effectiveness of our procedure.

### Candidate Genes in Haplotype-Blocks

Putative candidate genes in haplotype-blocks were identified by the following criteria: 1) SNP loci were identified that contain a low frequency allele (<0.1 estimated based on the Pool-Seq data) in the respective haplotype-block compared with the remaining base haplotypes and 2) for each haplotype-block genes were identified that contained at least one of the previously determined SNP loci using SNPeff version 3.3 (Cingolani et al. 2012) with the genome annotation dm5.48 (http://flybase.org/, last accessed April, 2014). This step was performed including and excluding 1,000-bp up/downstream regions and yielded identical gene sets in both cases.

## Supplementary Material

Supplementary material, scripts, figures S1–S16, and tables S1–S4 are available at *Molecular Biology and Evolution* online (http://www.mbe.oxfordjournals.org/).

Implemented scripts and the genome sequences of the 29 haplotypes of the experimental starting population and the 29 haplotypes of generation 67 are available from the Dryad Digital Repository under http://doi.org/10.5061/dryad.403b2.

Supplementary Data
