# Supplementary material for: Patterns of Linkage Disequilibrium and Long Range Hitchhiking in Evolving Experimental Drosophila melanogaster Populations
Source: Mol Biol Evol. 2014 Nov 17;32(2):495–509. doi: 10.1093/molbev/msu320 (PMC4298179; doi:10.1093/molbev/msu320)
Supplement: Supplementary Data [file supp_32_2_495__index.html]

Patterns of Linkage Disequilibrium and Long Range Hitchhiking in Evolving Experimental Drosophila melanogaster Populations — Patterns of Linkage Disequilibrium and Long Range Hitchhiking in Evolving Experimental Drosophila melanogaster Populations — Supplementary Data 

# Patterns of Linkage Disequilibrium and Long Range Hitchhiking in Evolving Experimental *Drosophila melanogaster* Populations

## Supplementary Data

files

**Files in this Data Supplement:**

- Supplementary Data - pdf file
- Supplementary Data - pdf file
- Supplementary Data - doc file
- Supplementary Data - xlsx file
