# Supplementary material for: Patterns of Linkage Disequilibrium and Long Range Hitchhiking in Evolving Experimental Drosophila melanogaster Populations
Source: Mol Biol Evol. 2014 Nov 17;32(2):495–509. doi: 10.1093/molbev/msu320 (PMC4298179; doi:10.1093/molbev/msu320)
Supplement: Supplementary Data [file supp_msu320_Supplementary_material.pdf]

Supplemental Material

Overview: **page**

Figure S1: Crossing scheme for the identification of full chromosomal haplotypes 2

Figure S2: Mean chromosomal coverage of F1 crosses to the reference strain 3

Figure S3: Haplotype coverage of the 58 full chromosomal haplotypes 4

Figure S4: Empirical false discovery rates (FDR) for haplotype base calls 5

Figure S5: Increase in short and long range LD within 67 generations 6

Figure S6: Evolved haplotypes from generation 67 for chromosomal arm 2L 7

Figure S7: Dependency of LD change after 67 generations from initial LD levels 8

Figure S8: Distribution of long range LD in the base and the evolved population (F67) 12

Figure S9: Decay of LD with physical distance for different categories 13

Figure S10: Identification of haplotype-blocks for all 10 base-haplotypes for which haplotype-blocks were identified (the figure can be found in a separate file.) 15

Figure S11 A,B: Visualization of each haplotype-block together with the evolved haplotypes from generation 67 (C-P can be found in a separate file.) 16

Figure S12: Allele sharing between haplotype-blocks suggests little recombination during 67 generations 17

Figure S13: Candidate ranks of haplo-block singleton markers over time 19

Figure S14: Frequency trajectories of haplotype-blocks covering the same genomic region 20

Figure S15: Accuracy in frequency estimation of base-haplotypes through haplotype specific singleton markers depending on the amount of known founder haplotypes 21

Table S1: Overview of haplotype-block identification in experimental and simulated data 22

Table S2: Overview of putative target SNPs and candidate genes in haplotype-blocks 22

Table S3: Overview of inversion breakpoints and low recombining genomic regions 22

Table S4: Accuracy in frequency estimation of base-haplotypes through haplotype specific singleton markers depending on the amount of known founder haplotypes 23

Overview of implemented scripts: 24

References: 25

Figure S1

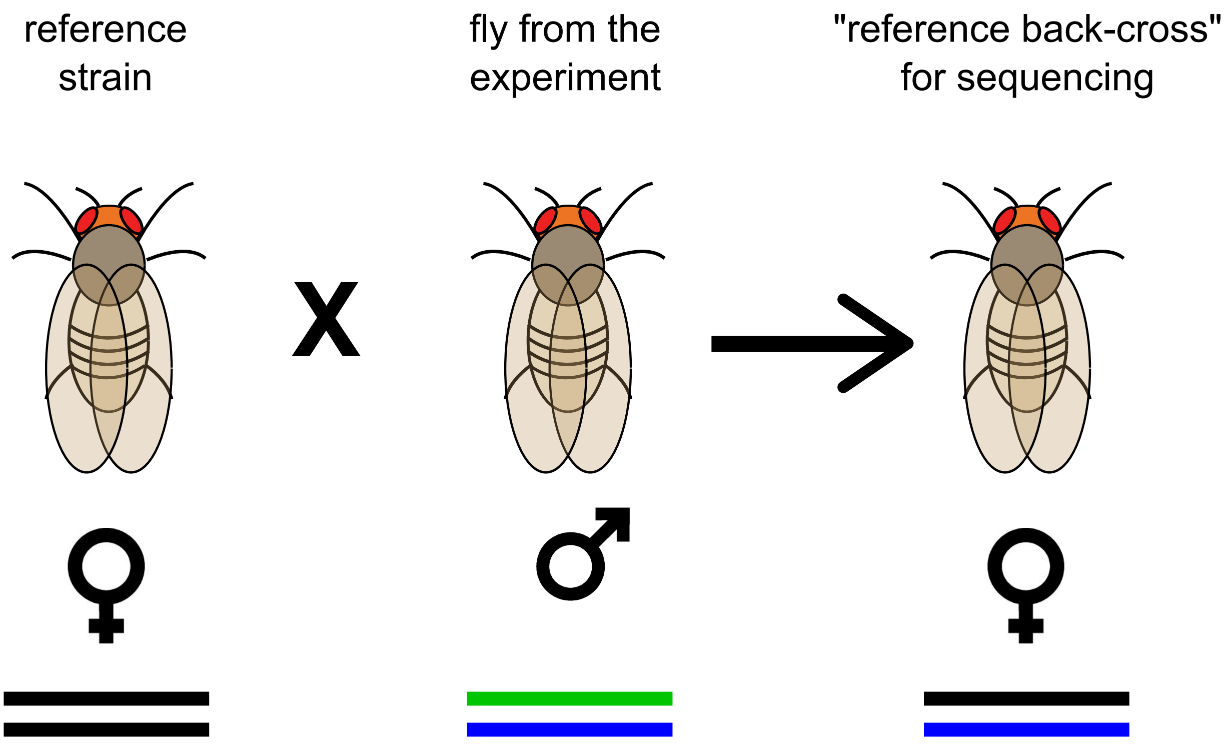

Fig. S1: Crossing scheme for the identification of full chromosomal haplotypes. A male fly is crossed to a virgin female of the inbred reference strain (y[1]; cn[1] bw[1] sp[1]). The female offspring of this “reference-cross” contains a full haploid chromosome set of the “experimental fly” as well as the reference strain. Bars depict the inheritance pattern of a chromosome. Note that this strategy is not informative for y-chromosomes, mtDNA and *Wolbachia*.

Figure S2

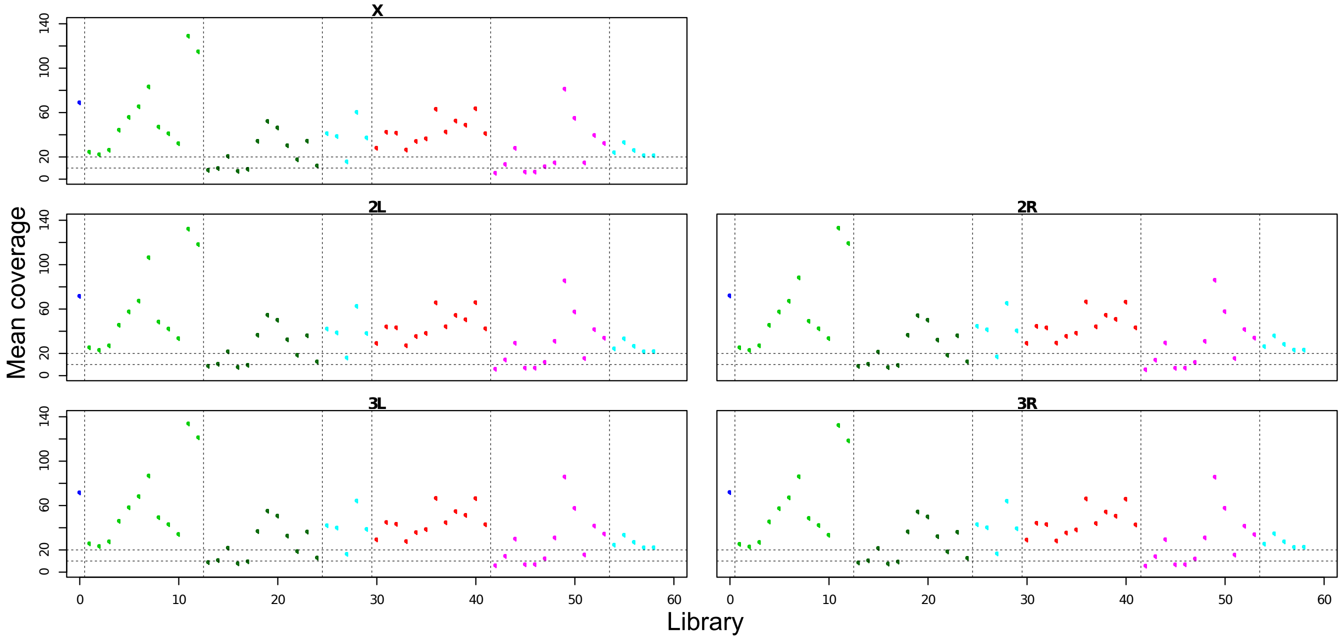

Fig. S2: Mean chromosomal coverage of F1 crosses to the reference strain. Sequenced flies (libraries 1-58) are F1 crosses of a fly from the experiment and the reference strain (y[1]; cn[1] bw[1] sp[1]). Libraries 1-29: F1 crosses to flies of the base population (F0, 29 different isofemale lines); libraries 30-58: F1 crosses to flies of the evolved population (F67, replicate R2). Colors represent different Illumina sequencing runs each with multiplexed samples. Library 0: 10 pooled individuals of the reference strain (Kapun *et al.* 2013).

Figure S3

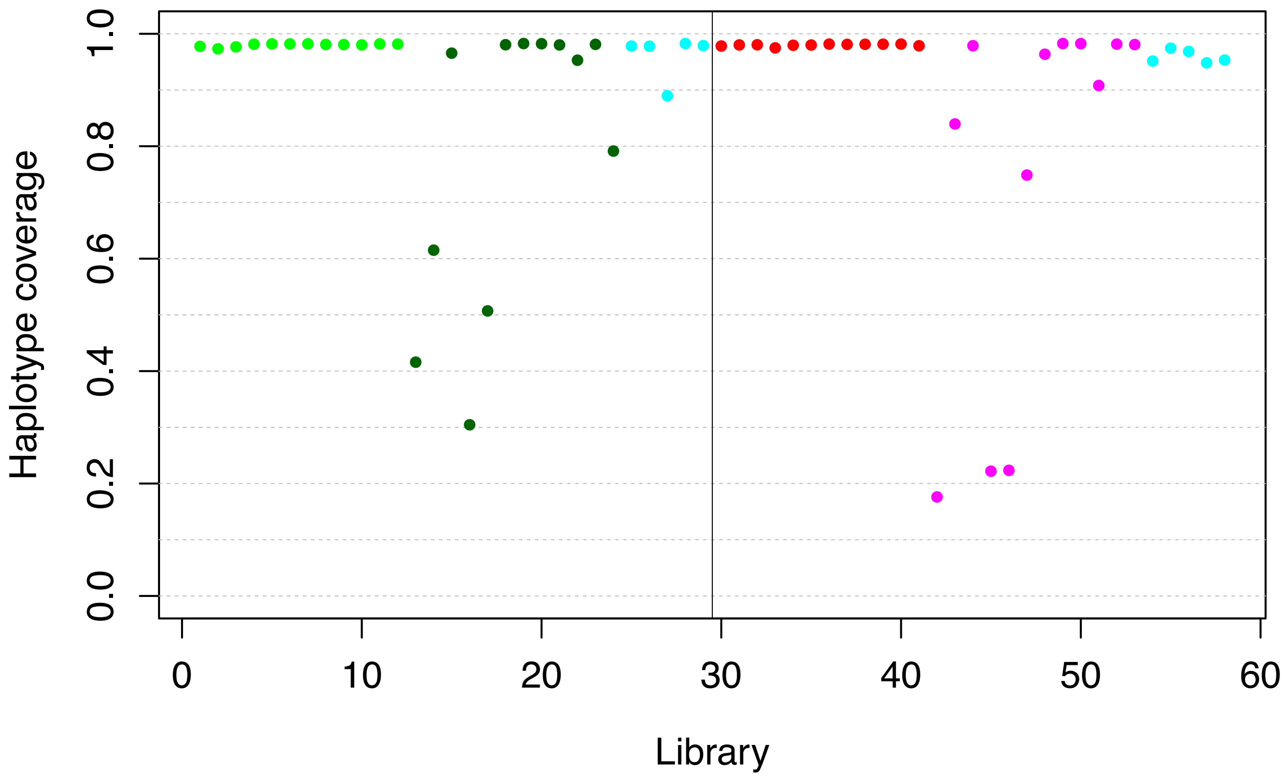

Fig. S3: Fraction of the genome for which haplotype information is available (haplotype coverage) for 58 full chromosomal haplotypes. Libraries 1-29: haplotype coverage of the base population (F0, 29 different isofemale lines); libraries 30-58: haplotype coverage of the evolved population (F67, replicate R2). Colors represent different Illumina sequencing runs with multiplexed samples. The maximal coverage equals ~105.8 Mb of the euchromatinic genome of *D. melanogaster*. 23 haplotypes of the base and 24 haplotypes of the evolved population exceed a haplotype coverage of 90%.

Figure S4

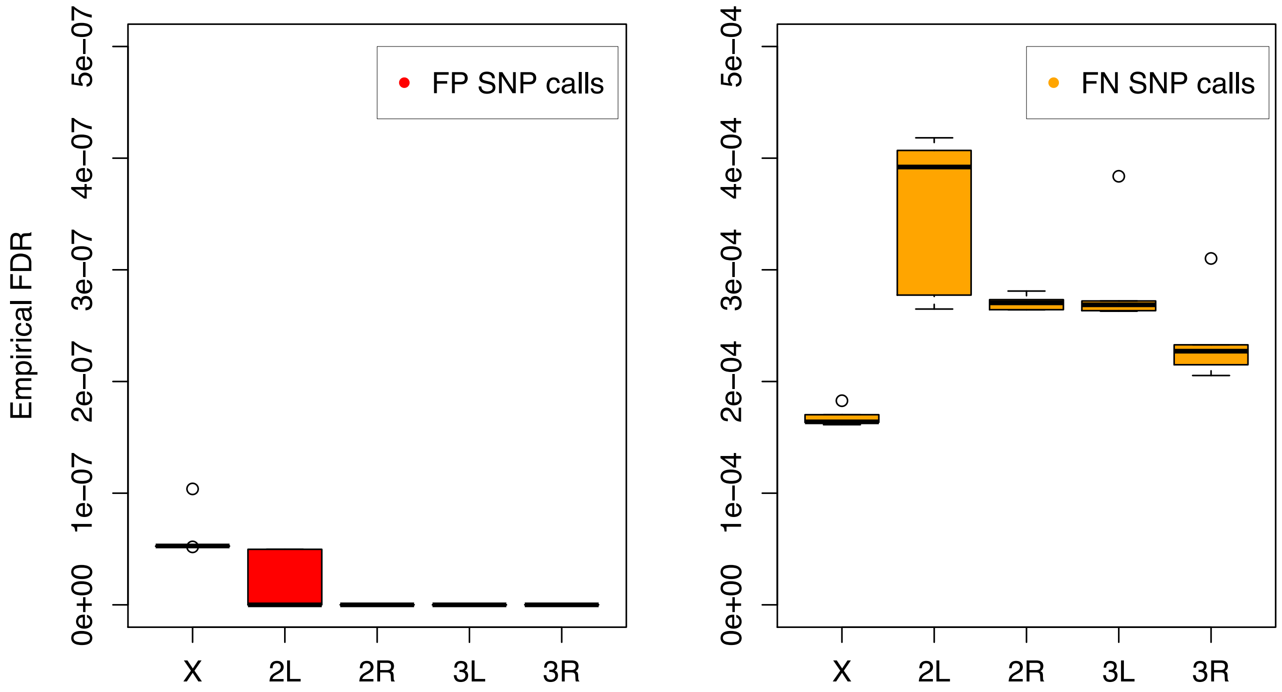

Fig. S4: Empirical false discovery rates (FDR) for haplotype base calls. FDR were determined for chromosomal regions with a low sequencing coverage. Assuming higher coverage more likely identifies the true SNP, 5 highly covered libraries (> 60 fold mean coverage; libraries 7, 11, 12, 36, 40) were down-sampled to a coverage of 10. Base calls were compared between full and down-sampled libraries and false positive (FP) SNP calls (SNP calls in the down-sampled but not in the full library) and false negative (FN) SNP calls (SNP calls in the full but not in the down-sampled library) were determined. Boxes represent FDR estimates from the 5 different libraries. FDR for low coverage regions is generally low $<=10^{-7}$ for FP and <0.0005 for FN SNP calls with respect to the reference.

Figure S5

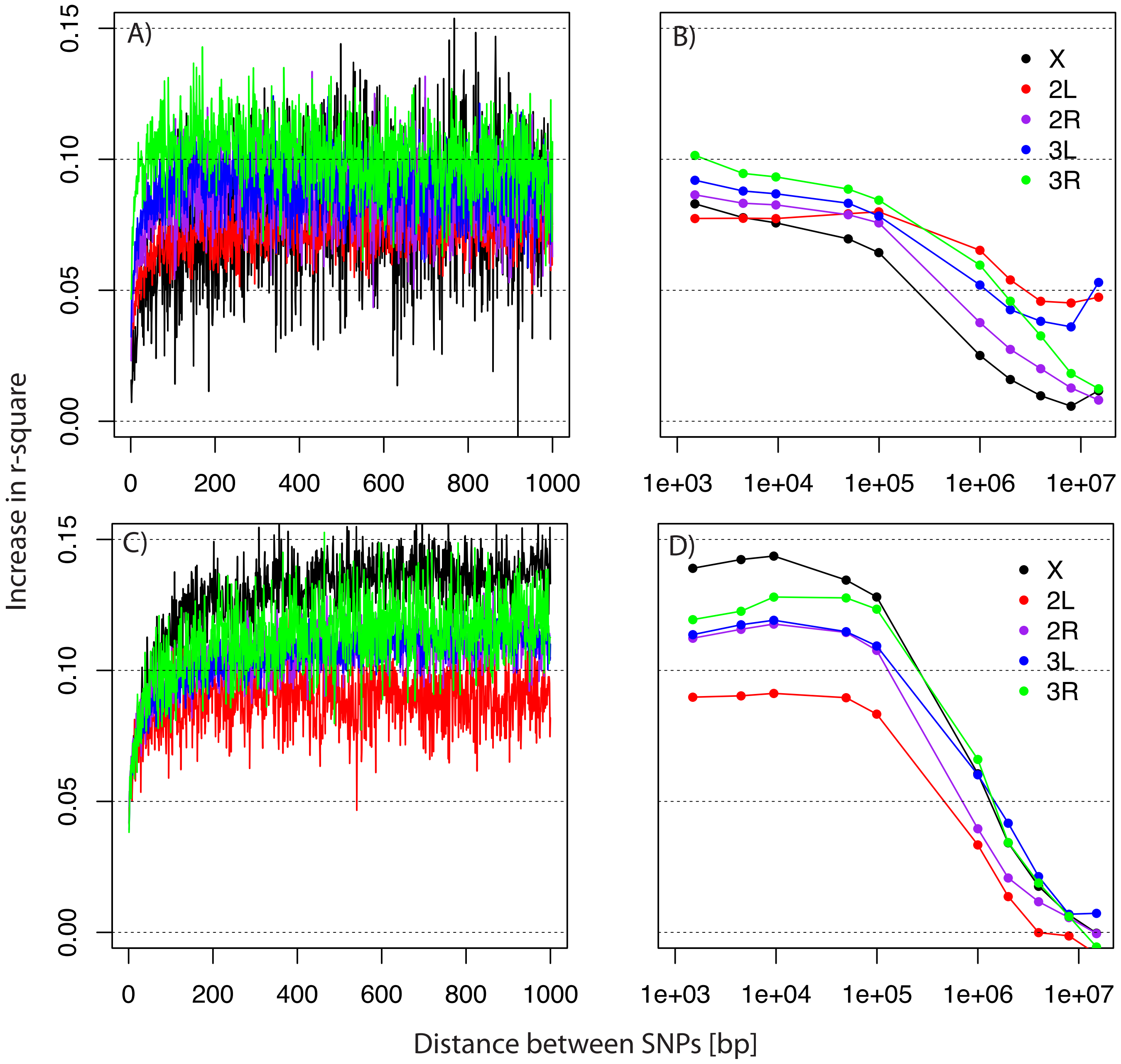

Fig. S5: Increase in short and long range LD within 67 generations for experimental and simulated data. Increase in mean LD is shown across all SNP-pairs with the respective physical distance [in bp] for the experimental A&B and the simulated C&D data (see Material & Methods for the simulation parameters). LD estimates (r^2) are based on SNPs that are polymorphic in the base and the evolved population, with at least 24 haplotypes. B&D) Data points for long range LD estimates (full circles) present averages of 1 Kb windows for distances of 2 kb, 5 kb, 10 kb, 50 kb, 100 kb, 1 Mb, 2Mb, 4Mb, 8 Mb, 15 Mb. Increase in mean LD is constant in the short range and slowly drops with increasing distance > 10 kb in both data sets. The slightly stronger increase in the short range in the simulated data suggests that the simulated N_e of 200 underestimates the N_e in the experiment over 67 generations. Moreover, the slower drop of LD in the long range in the experimental data suggests the influence of features not present in the simulations such as large inversions and/or (strong) selection.

Figure S6:

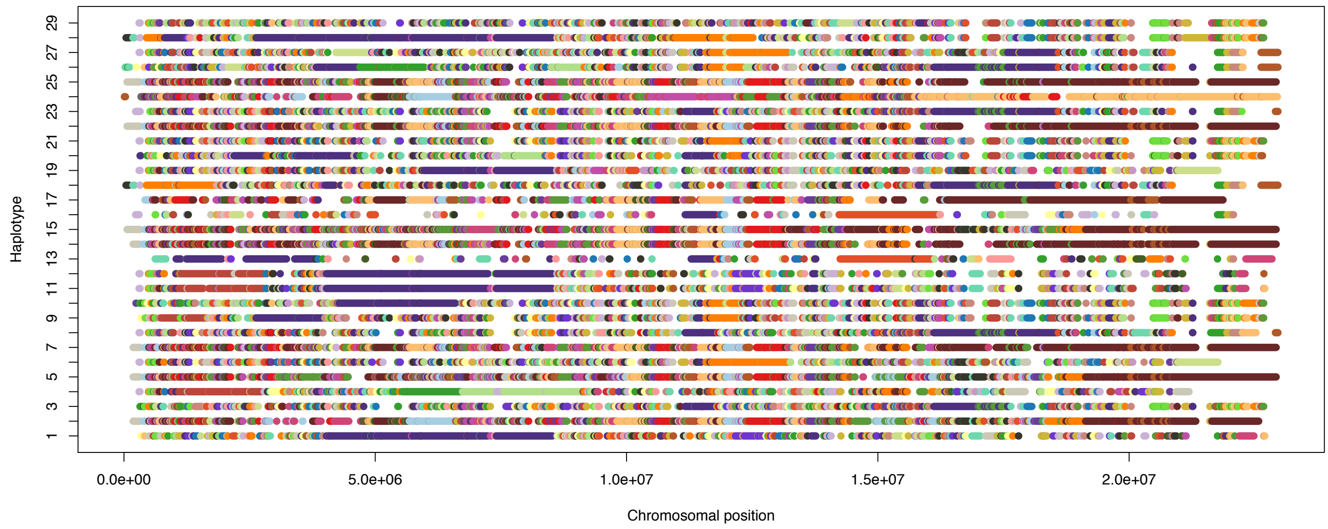

Fig. S6: Evolved haplotypes from generation 67 for chromosomal arm 2L. The haplotype-block structure is presented. Each row corresponds to one evolved haplotype. Base-singletons called from 29 base-haplotypes (a subset of ~113 haplotypes present in the experimental base population) specific to the respective base-haplotype have been used to infer presence of base-haplotype fragments in the evolved haplotypes and are painted in different colors. Only marker alleles were used if at least two markers of the same base-haplotype were adjacent to each other (single marker alleles surrounded by markers alleles from different base-haplotypes were omitted as most likely to be sequencing of marker calling errors).

The interpretation of this visualization is complicated by the inaccuracy of initial marker calling from a subset of the base population. In some regions large fragments of initial base-haplotypes can be seen, which is in accordance with the observed increase in long range LD (Fig. 1. Fig. S5) and supports our claims of kb to Mb sized block moving in our experiment. However, it is also expected that the evolved haplotypes are mosaics with a large fraction of the ~84 unknown base-haplotypes. As the unknown base-haplotypes were not included for marker calling of the 29 known base-haplotypes it is likely that haplotype markers contain a considerable fraction that are not unique to the intended base-haplotype but also present on an unknown base-haplotype. Unknown founder haplotypes are therefore partly visualized through the non-presence of haplotype markers or as largely seen here via very fine mosaics of the 29 different base-haplotype markers. This explanation is also strongly supported when zooming into smaller genomic regions (data not shown).

Figure S7

A) Chromosome arm 2L

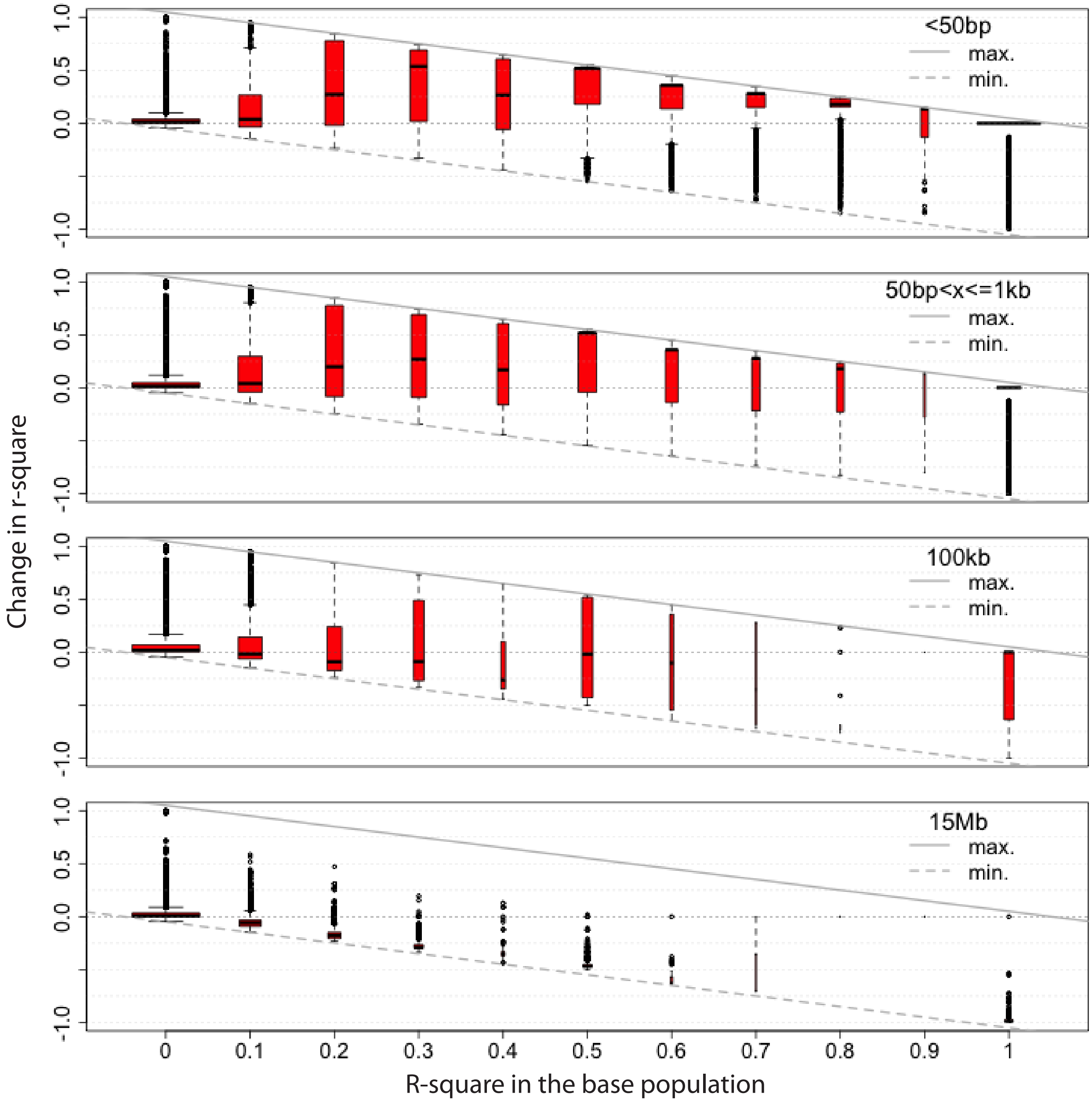

B) Chromosome arm 2R

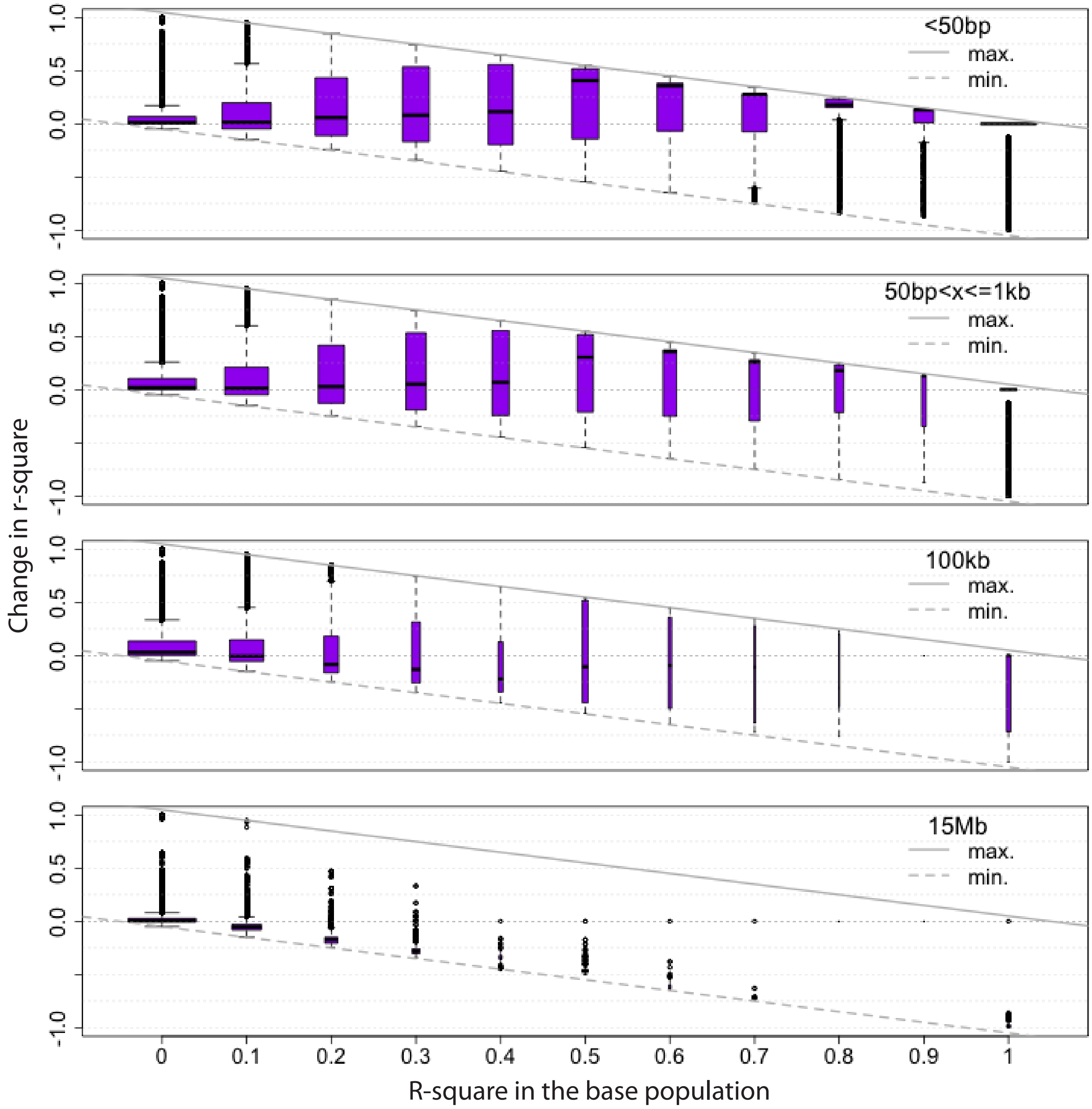

C) Chromosome arm 3L

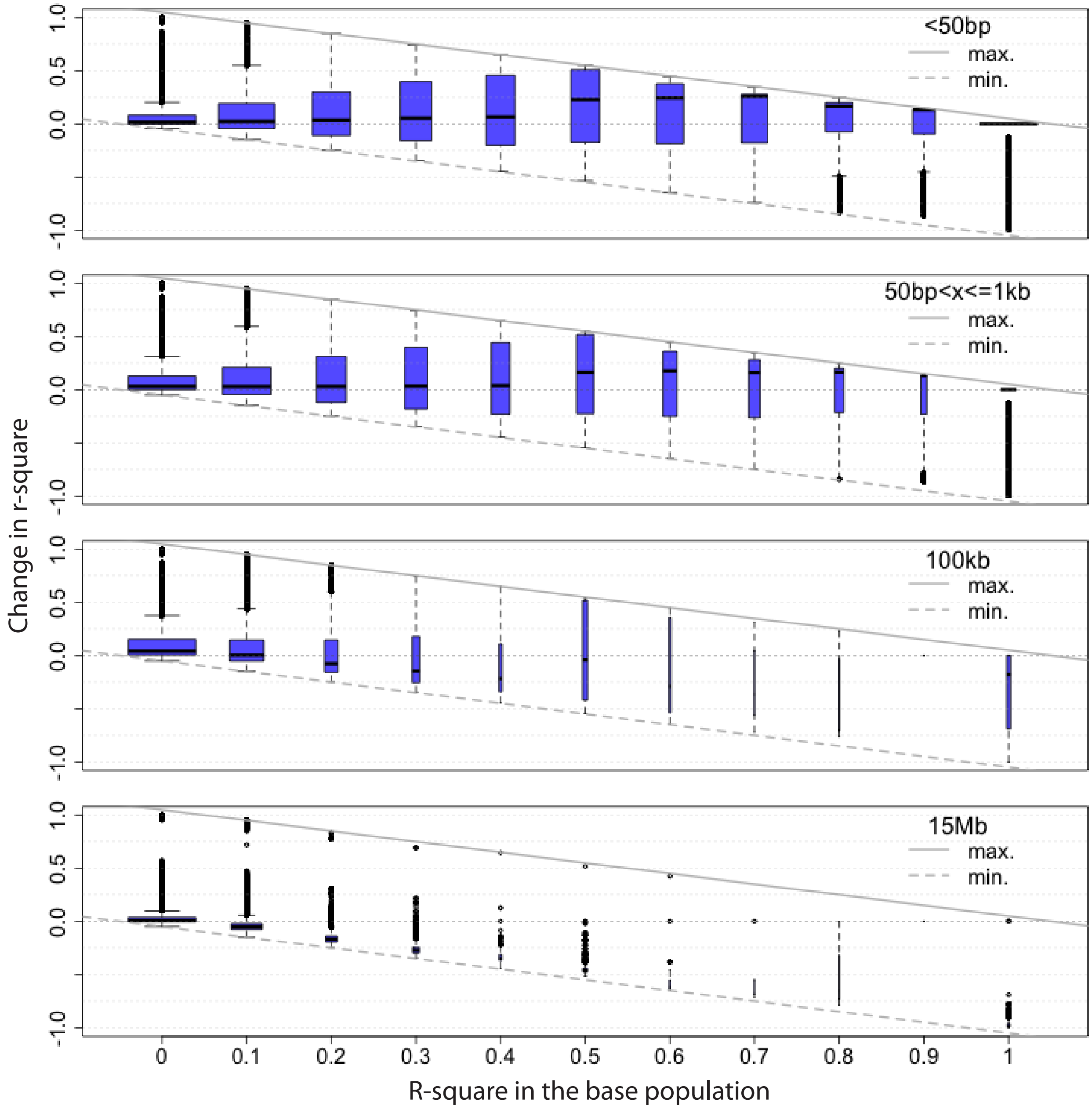

D) Chromosome X

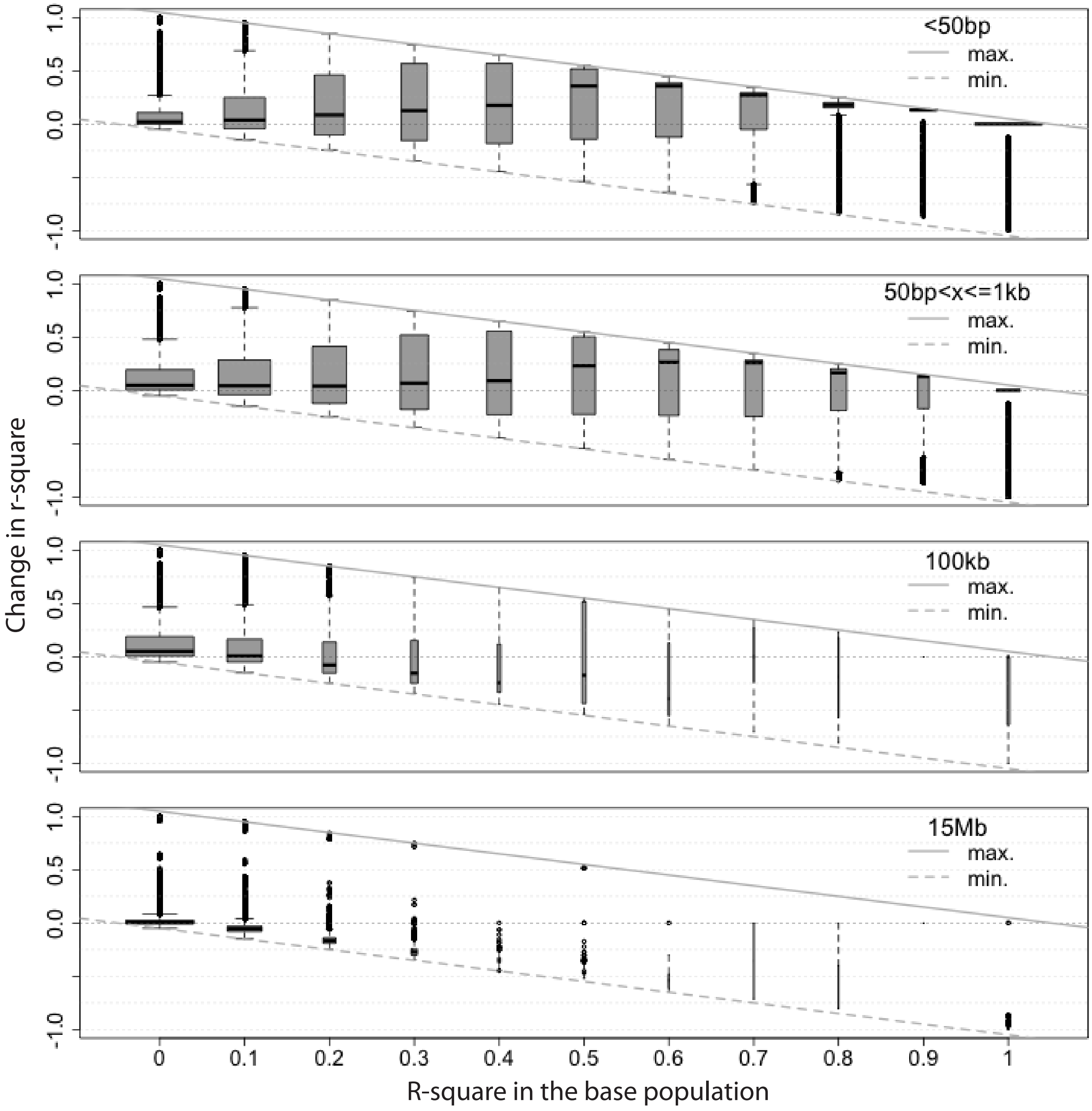

Fig. S7: Change in linkage disequilibrium after 67 generations of evolution depends on the initial LD levels. A-D) Results for the different chromosome arms 2L (red), 2R (purple), 3L (blue) and X (gray). Boxplots indicate LD change after 67 generations (y-axis) for different categories of initial LD values (x-axis). In each row SNPs with different spacing are shown: <=50 bp (1st row), 51 bp to 1 kb (2nd row), 99 kb to 100 kb (3rd row) and 14.009 Mb to15 Mb (4th row). Solid and dashed gray lines indicate the maximum, minimum possible change in LD, respectively. Width of the boxes is scaled by category size (i.e.: number of pairwise comparisons). r^2 was calculated for matched SNPs polymorphic in both populations.

Across all chromosome arms the following patterns are present: 1) In the short range (<=1kb), where recombination events are very rare, LD increase is more likely if initial LD is present. 2) If new recombination events are more frequent (distances ~100kb) LD increase is no longer facilitated by initial LD; LD in the starting population typically decreases for large distances when new recombination events are frequent (~15Mb). 3) For the majority of SNPs, r^2 takes extreme values (close to either zero or one). 4) Over short distances most SNPs experience change in LD since initial LD is high and recombination during the experiment can be neglected.

Figure S8

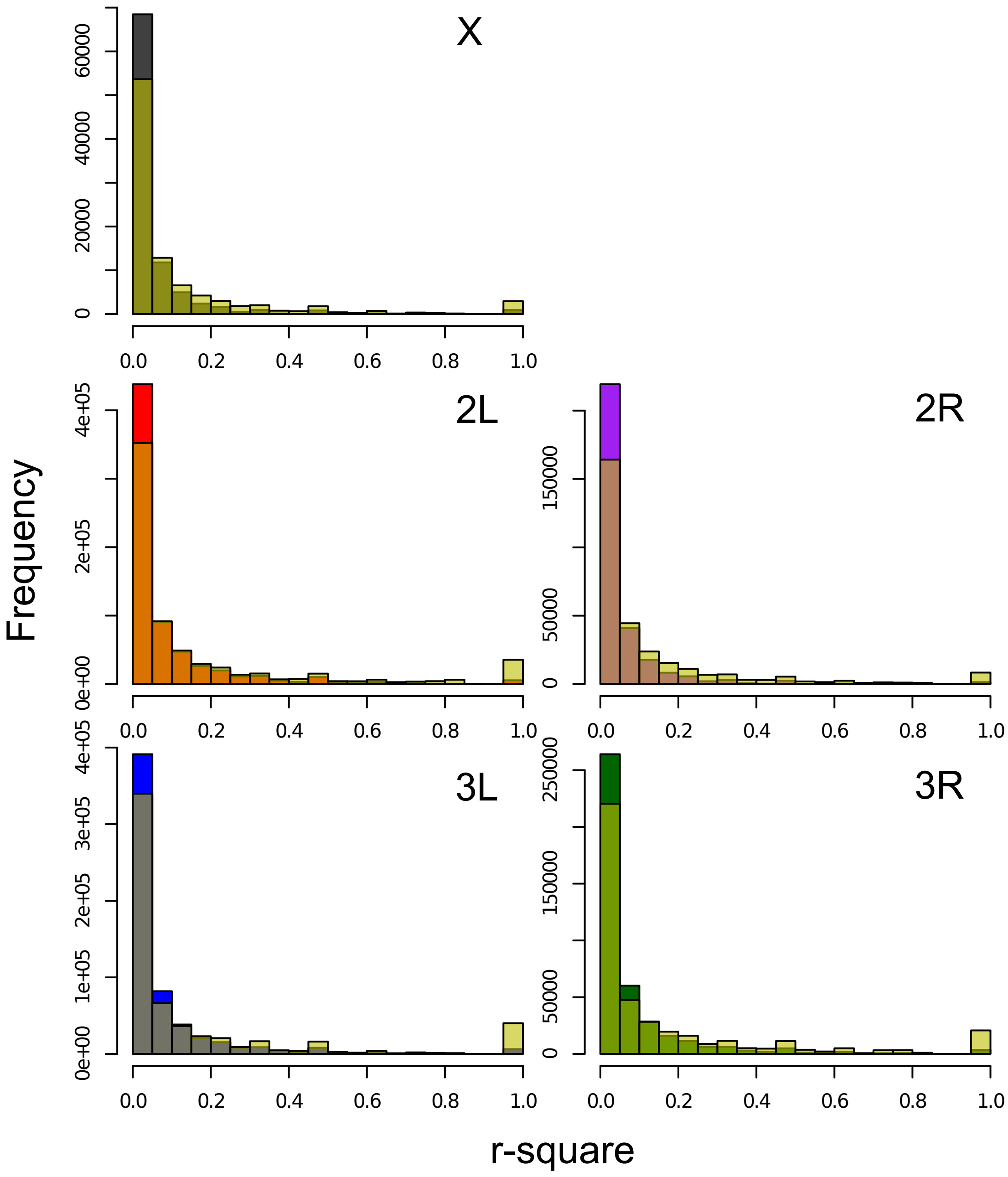

Fig. S8: Distribution of long range LD in the base and the evolved population (F67). Overlapping histograms of LD for the base (black: X, red: 2L, purple: 2R, blue: 3L, green: 3R) and the evolved population (yellow). The intermediate color in each plot visualizes the overlap between both populations. Long range LD is shown for distances of 99 kb to 100 kb, only for matched SNP-pairs polymorphic in both populations. Increase in long range LD in the evolved population is caused by a relatively small fraction of SNP-pairs with very high LD (r^2=1).

Figure S9

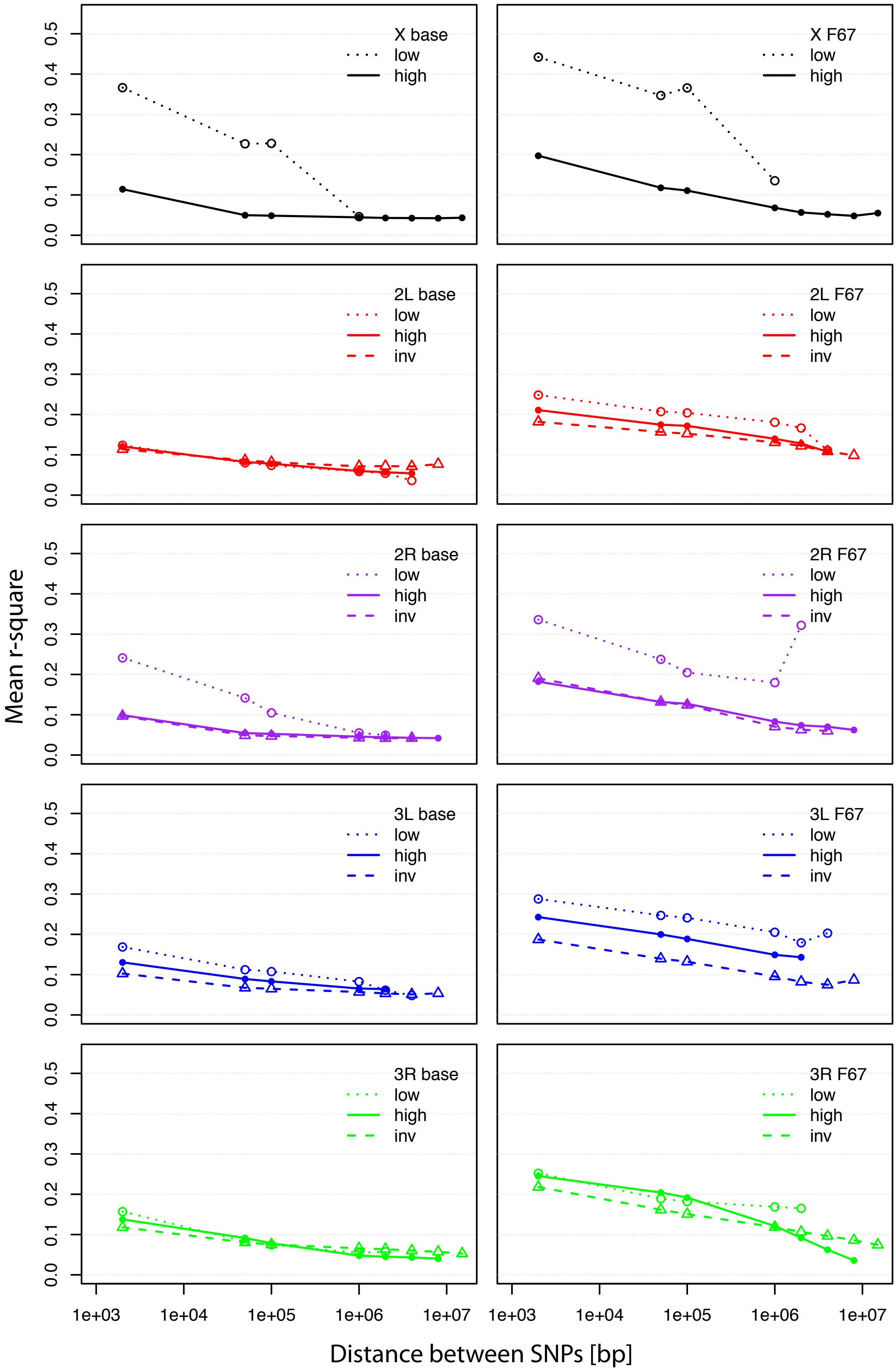

Fig. S9: Decay of long range LD with physical distance in the base and the evolved (F67) population for three different categories of genomic regions: 1) low recombining regions (dotted lines and open circles), 2) genomic regions covered by cosmopolitan inversions segregating in our populations (Table 1) (dashed lines and open triangles); and 3) the remaining high recombining regions (solid lines and full circles). LD

estimates (r^2) are based on SNPs that are polymorphic in the base and the evolved population, with a minimum of 24 haplotypes. Data points for long range LD estimates (dots) present averages of 1 kb windows for distances of 2 kb, 50 kb, 100 kb, 1 Mb, 2Mb, 4Mb, 8 Mb, 15 Mb. r^2 is higher in the low recombining than the high recombining regions in the base population for X and 2R and for most chromosome arms at generation F67. Inversion regions show similar or reduced amounts of LD compared to the high recombining regions, particularly in the evolved population.

Figure S10

Fig S10: Identification of haplotype-blocks for all 10 base-haplotypes for which haplotype-blocks were identified. Panels 1-10 correspond to base haplotypes b1, b5, b6, b7, b12, b17, b19, b21, b24 and b26. Singletons specific to the respective haplotype were identified from the 29 base haplotypes, grouped into windows of 20 (overlap 15). For each singleton-window the frequency changes (inferred from Pool-Seq) are plotted for three different comparisons of the base to generation F15 (green), F37 (blue) and F59 (red). Dashed lines represent thresholds for haplotype-block identification for the respective generations based on mean frequency changes of the top 2,000 CMH candidates (see Material & Methods). Short solid colored lines above present areas of identified haplotype-blocks. The ten panels show haplotype-block identification through frequency changes of base specific singleton-SNPs.

The figure is provided as a separate supplemental pdf file to be able to provide a better resolution.

Figure S11

A) haplotype-block 9

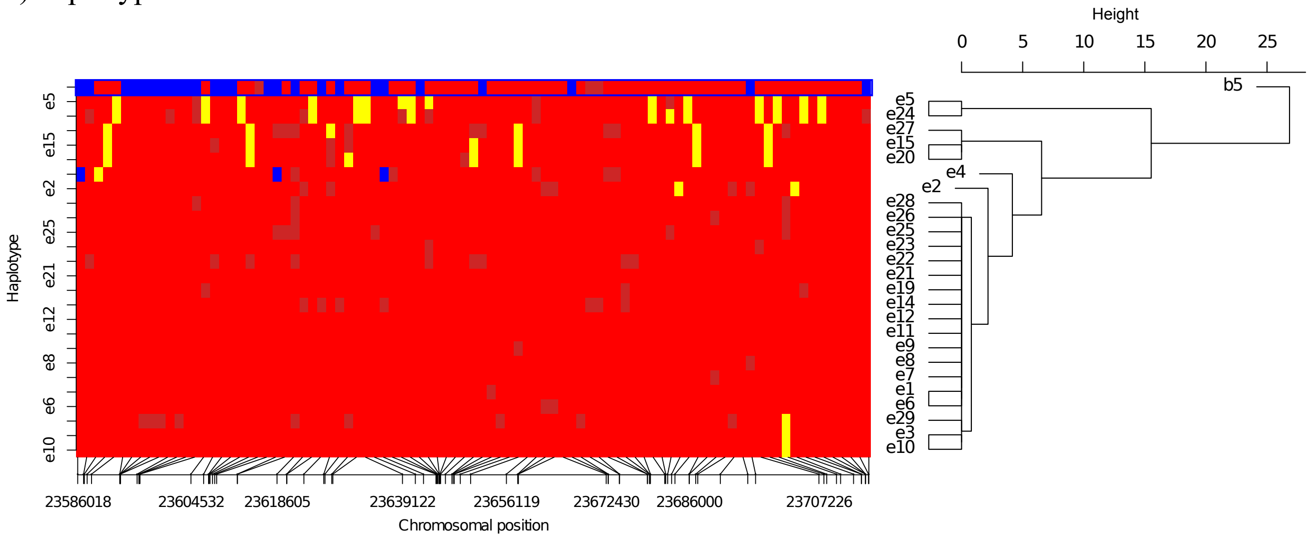

B) haplotype-block 11

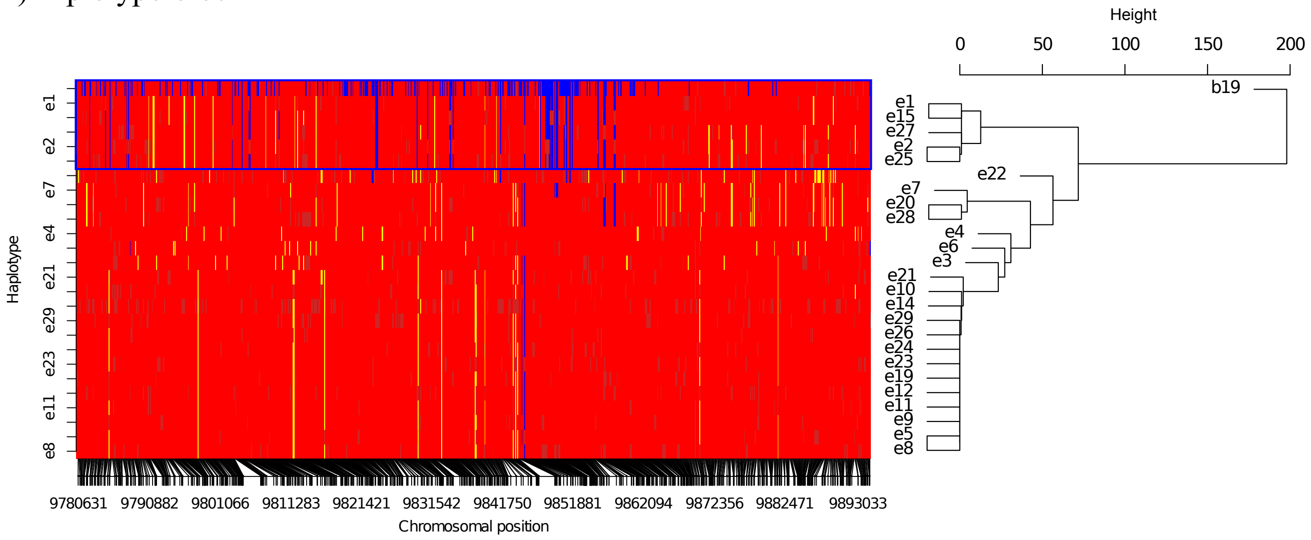

Fig. S11: Evolved haplotypes from generation 67 with each haplotype-block from the base population, respectively. A) haplotype-block 9, B) haplotype-block 11, C-P) haplotype-blocks 1-8, 10, 13-17. Haplotype-blocks from the base population are labeled with b, evolved haplotypes are labeled with e. The left panel represents the haplotype block structure. Each horizontal line corresponds to one haplotype. Base-singletons specific to the respective haplotype-block are indicated in blue, yellow indicates the remaining singletons in the base population. The major allele in the base population is shown in red. Missing data are shown in grey. Only loci that are singletons for any base-haplotype are shown. The right panel shows a cladogram based on singleton-SNP sharing. The blue rectangle marks the evolved haplotypes that cluster with the respective haplotype-block sequence from the base population. If present blue rectangles in dashed lines highlights parts that also might have originated from the original haplotype-block sequence. For different haplotype-blocks its core is shared to different extends with the evolved haplotypes that cluster around it. (Parts C-P are shown in a separate file; figure S11 also shown in a separate file uses an alternative visualization for the same 17 haplotype-blocks.)

Figure S12

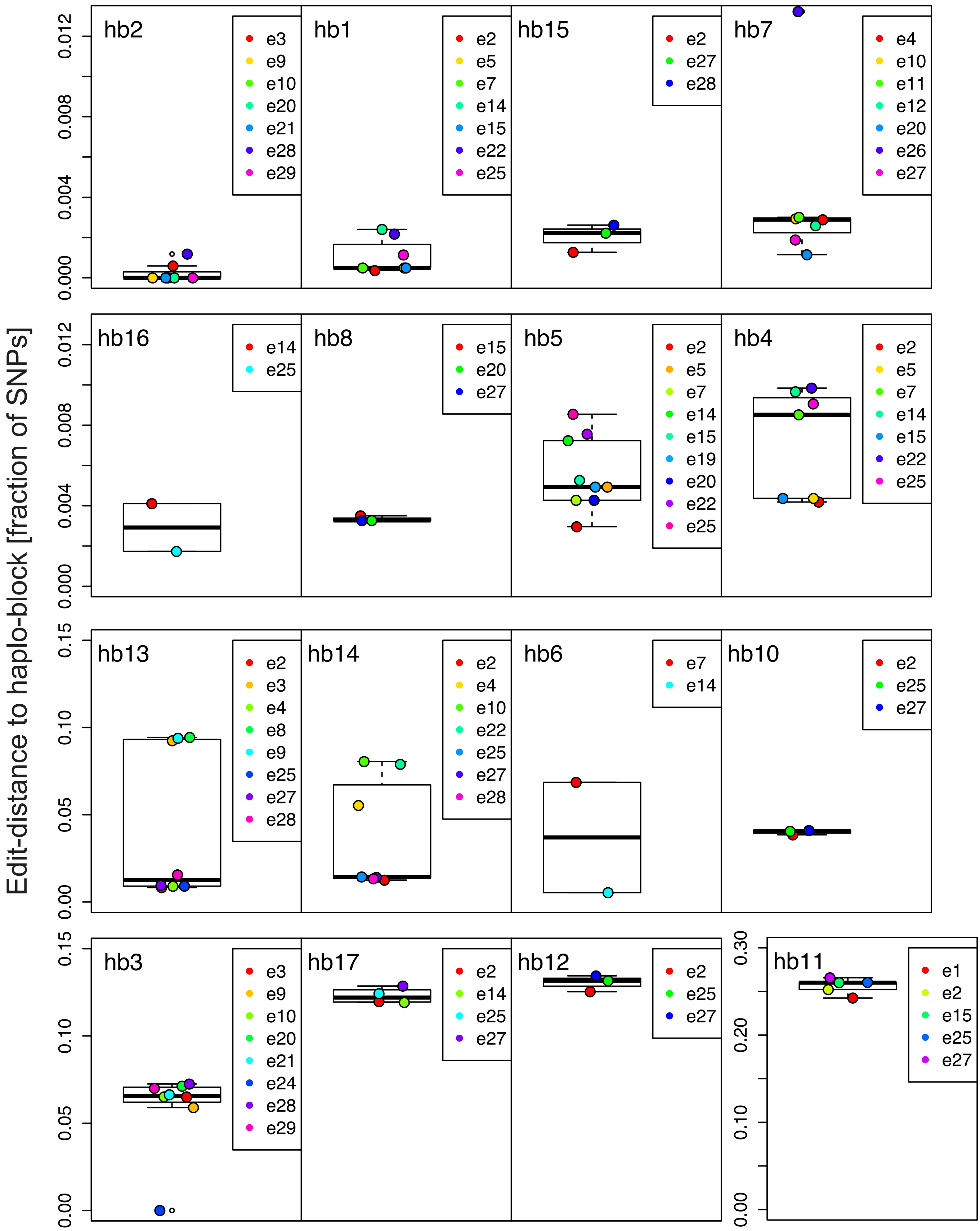
Edit-distance to haplo-block [fraction of SNPs]
hb2
e3
e9
e10
e20
e21
e28
e29
hb1
e2
e5
e7
e14
e15
e22
e25
hb15
e2
e27
e28
hb7
e4
e10
e11
e12
e20
e26
e27
hb16
e14
e25
hb8
e15
e20
e27
hb5
e2
e5
e7
e14
e15
e19
e20
e22
e25
hb4
e2
e5
e7
e14
e15
e22
e25
hb13
e2
e3
e4
e8
e9
e25
e27
e28
hb14
e2
e4
e10
e22
e25
e27
e28
hb6
e7
e14
hb10
e2
e25
e27
hb3
e3
e9
e10
e20
e21
e24
e28
e29
hb17
e2
e14
e25
e27
hb12
e2
e25
e27
hb11
e1
e2
e15
e25
e27
0.000
0.004
0.008
0.012
0.00
0.05
0.10
0.15
0.00
0.10
0.20
0.30

Fig. S12: Allele sharing between haplotype-blocks suggests little recombination during 67 generations. Each plot shows the amount of allele sharing between a haplotype-block and the corresponding evolved haplotypes of generation F67 (identified via Fig. 7, Fig. S10). Allele sharing is calculated as average pair-wise distance in the complete region. Colored points on top of the boxplots indicate the evolved haplotypes. Haplotype-block plots are sorted in ascending order with respect to the divergence of the evolved haplotypes (note that we used three different scales on the y-axis). Evolved haplotypes carrying the haplotype-block sequences show small but variable amounts of recombination during 67 generations of experimental evolution.

Figure S13

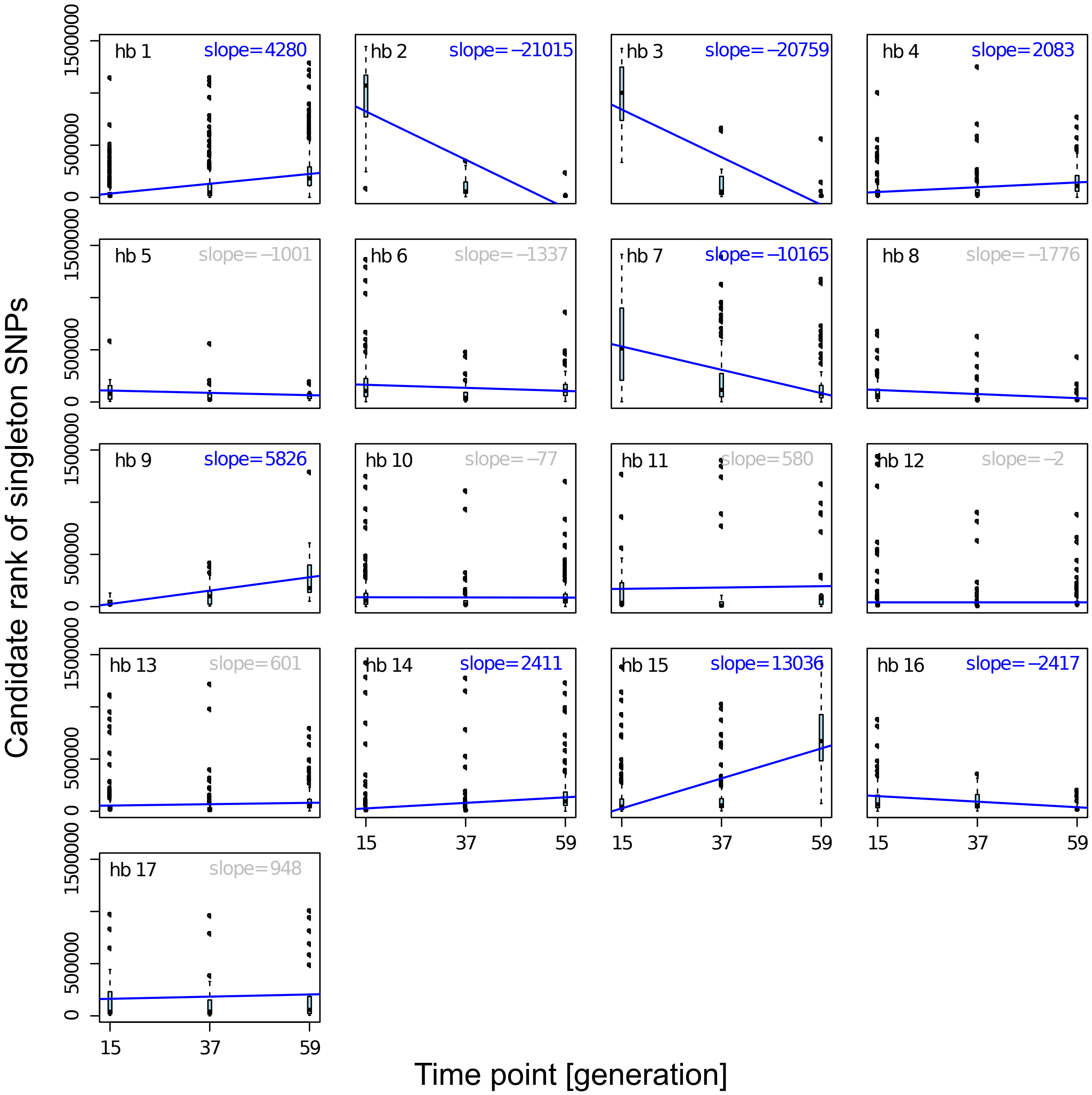

Fig. S13: Candidate ranks of haplo-block singleton markers over time. Each plot shows the rank of all singleton-SNPs for the respective haplotype-block. Ranks for each time point were computed via the CMH test for the comparison base to the respective time point. Blue lines indicate linear regression lines. Slope values for significant differentiations from zero are plotted in blue, otherwise gray (Linear Model: rank ~ time point, FDR<0.001; FDR values for haplotype-blocks 1-17: 1.74e-63, 1.05e-16, 5.27e-13, 1.46e-07, 1, 0.224, 6.42e-41, 1.19e-02, 4.50e-04, 1, 1, 1, 2.245e-01, 3.21e-09, 3.44e-55, 5.23e-05, 1). Five haplotype-blocks show decreasing candidate enrichment over time, while 4 blocks show an increasing candidate enrichment over time.

Figure S14

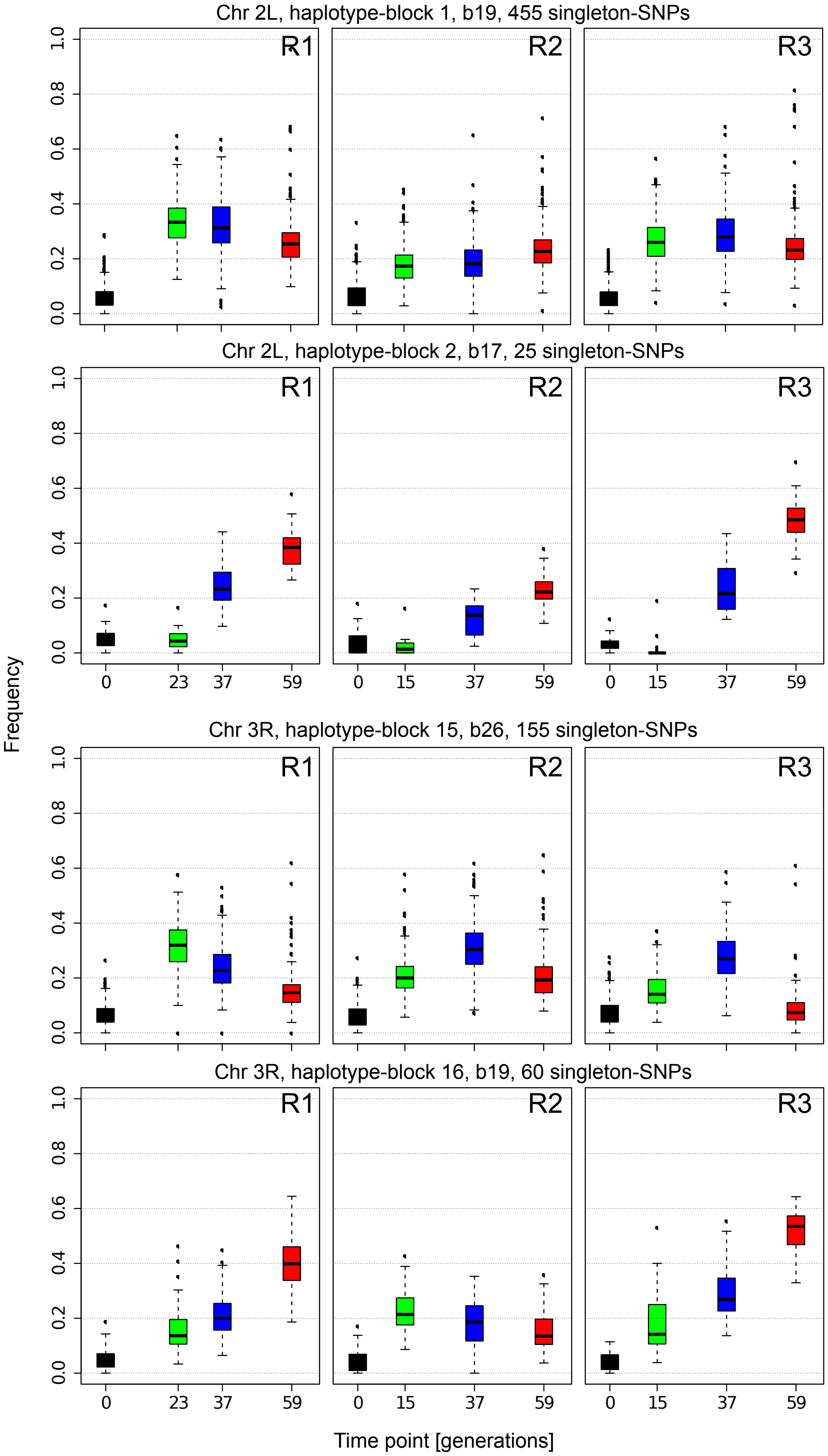

Fig. S14: Frequency trajectories of overlapping haplotype-blocks. The first two panels show haplotype-block trajectories of blocks 1 & 2 overlapping near the centromere of chromosome 2L; the last two panels show trajectories of the haplotype-blocks 15 & 16 overlapping on chromosome arm 3R. Trajectories are shown for all three replicates (R1, R2, R3). Frequencies are estimated by haplotype specific SNP-markers in the base population. Haplotype-blocks covering the same genomic region often show inverse dynamics over time.

Figure 15

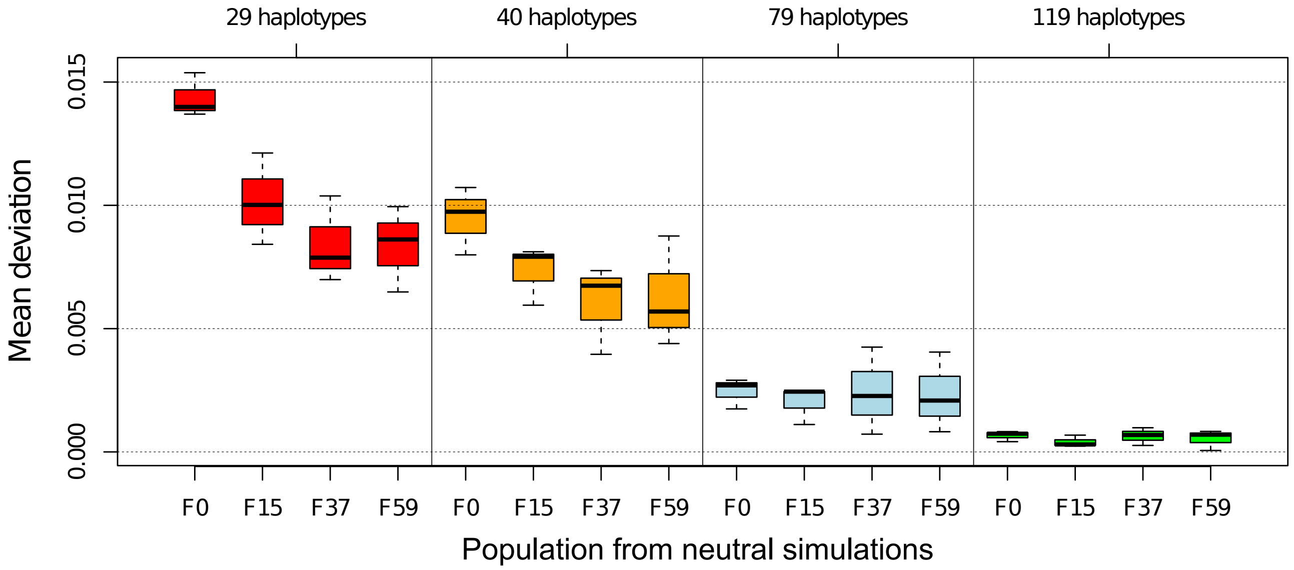

Fig. 15: Accuracy in frequency estimation of base-haplotypes through haplotype specific singleton markers depending on the amount of known founder haplotypes. Haplotypes frequencies were calculated for sliding windows of 20 haplotype singleton markers through median marker frequencies based on subsets of 29, 40, 79 and 119 out of 158 base-haplotypes corresponding to 18%, 20%, 50% and 75% of known base haplotypes. The mean deviation between estimated and true frequencies was calculated based on 4,000 – 11.000 windows per chromosomal arm (Table S4). Boxplots display results for chromosomal arms 2L, 3L and X. Deviations were calculated for haplotype compositions obtained from neutral simulations with Ne=200 and no recombination from a starting population of equal amounts of 158 DGRP (Mackay *et al.* 2012) haplotypes with MimicrEE (Kofler & Schlötterer 2013). Mean deviations in frequency estimated for 20 marker haplotype windows decrease with the amount of known base haplotypes.

Table S1: Overview of haplotype-block identification in experimental and simulated data. "Exp" refers to the experimental data. Sim1-Sim5 refers to independent neutral simulations. Sim5a and Sim5b describe the identical simulation, which was analyzed with a different random set of 29 base-haplotypes. Singleton-SNP windows (sSNP windows) indicates how many singleton-SNP windows exceeded the outlier threshold for the respective time point.

ID	Base population	N_e *1	sSNP windows in haplotype-blocks (F15)	sSNP windows in haplotype-blocks (F37)	sSNP windows in haplotype-blocks (F59)	Total number of sSNP windows in haplotype-blocks
Exp	113 isofemale lines	~183*	154	230	13	301
Sim1	DGRP *2	250	0	0	0	
Sim2	DGRP *2	250	0	0	0	
Sim3	Simulated-base *3	250	0	0	0	
Sim4	DGRP *2	150	10	0	0	10
Sim5a	DGRP *2	150	0	0	0	
Sim5b	DGRP *2	150	0	0	0	

*1 N_e was estimated by Tobler *et al.* (2013) based on changes in the site frequency spectrum between the base and F15 for the X chromosome.
*2 Full chromosome haplotype sequences from Mackay *et al.* (2012)
*3 Full chromosome haplotype sequences from Bastide *et al.* (2013)

Table S2: Overview of candidate genes in all haplotype-blocks and putative target SNPs in haplotype-block 9.

This table is provided as an additional excel file.

Table S3: Overview of inversion breakpoints and low recombining genomic regions. Remaining regions are defined as high recombining.

Chromosome arm	inversion			proximal low recombination region		distal low recombination region	
		breakpoint 1	breakpoint 2	border 1	border 2	border 1	border 2
X		-	-	0	1,200,000	21,300,000	22,422,827
2L	*In(2L)t*	2,225,744	13,154,180	0	500,000	18,800,000	23,011,544
2R	*IN(2R)Ns*	11,278,659	16,163,839	0	2,200,000	20,900,000	21,146,708
3L	*In(3L)P*	3,173,046	1,630,194	0	800,000	19,000,000	24,543,557
3R	*In(3R)K*	7,576,289	21,966,092	0	3,800,000	27,500,000	27,905,053
	In(3R)Payne	12,257,931	20,569,732	-	-	-	-
	In(3R)C	15,922,589	28,234,649	-	-	-	-
	In(3R)Mo	17,232,639	24,857,019	-	-	-	-

Inversion breakpoints are taken from Corbett-Detig *et al.* (2013) apart from In(3R)c taken from Ashburner & Lemeunier (1976). Low recombination regions are defined as regions with 0 cM/100 kb (Fiston-Lavier *et al.* 2010).

Table S4: Accuracy in frequency estimation of base-haplotypes through haplotype specific singleton markers depending on the amount of known founder haplotypes. Haplotypes frequencies were calculated for sliding windows of 20 haplotype singleton markers through median marker frequencies based on subsets of 29, 40, 79 and 119 out of 158 base-haplotypes corresponding to 18%, 20%, 50% and 75% of known base haplotypes. The mean deviation between estimated and true frequencies was calculated based on 4,000 – 11.000 windows per chromosomal arm (sample size). Results are shown for chromosomal arms 2L, 3L and X. Deviations were calculated for haplotype compositions obtained from neutral simulations with Ne=200 and no recombination from a starting population of equal amounts of 158 DGRP (Mackay *et al.* 2012) haplotypes with MimicrEE (Kofler & Schlötterer 2013). Mean deviations in frequency estimated for 20 marker haplotype windows decrease with the amount of known base haplotypes.

	mean deviation of frequency estimates			
	# haplotypes and % of base-haplotypes (158)			
	29, 18%	40, 25%	79, 50%	119, 75%
2L				
base population	0.0140	0.0097	0.0029	0.0008
generation 15	0.0100	0.0079	0.0024	0.0007
generation 37	0.0079	0.0074	0.0043	0.0010
generation 59	0.0086	0.0054	0.0040	0.0007
sample size (# windows)	7440	8232	9223	9917
3L				
base population	0.0154	0.0107	0.0027	0.0007
generation 15	0.0121	0.0081	0.0025	0.0002
generation 37	0.0104	0.0067	0.0023	0.0003
generation 59	0.0099	0.0088	0.0021	0.0001
sample size (# windows)	8046	8089	9969	10774
X				
base population	0.0137	0.0080	0.0017	0.0004
generation 15	0.0084	0.0060	0.0011	0.0003
generation 37	0.0070	0.0040	0.0007	0.0007
generation 59	0.0065	0.0044	0.0008	0.0008
sample size (# windows)	4425	4750	6037	6911

Overview of implemented scripts

1) Estimation of short and long range LD from haplotype data

Script 1: calc_r2_min-maxdist.py

Calculation of r^2 values from whole genome haplotype data: r^2 estimation for all SNP-pairs on a chromosome with physical distances within the provided range.

2) Identification of haplotype-blocks

Script 2: haplotype_singleton-blocks_window_EE_repl.py

Estimation of frequency changes of base-haplotypes between the base and subsequent time points: frequency changes of base haplotypes are estimated via haplotype specific singletons in the base population. Besides knowledge of haplotype specific singletons, the singleton allele frequencies should be known for at least two replicates in the base population and subsequent time points during experimental evolution.

Haplotype-blocks have to be called separately afterwards using time point specific thresholds (for required frequency changes) and windows have to be combined if they overlap or are within a defined recombination distance.

References

Ashburner M, Lemeunier F (1976) Relationships within the *melanogaster* Species Subgroup of the Genus *Drosophila* (Sophophora). I. Inversion Polymorphisms in *Drosophila melanogaster* and *Drosophila simulans*. *Proceedings of the Royal Society of London. Series B, Biological Sciences*, **193**, 137–157.

Bastide H, Betancourt A, Nolte V *et al.* (2013) A Genome-Wide, Fine-Scale Map of Natural Pigmentation Variation in *Drosophila melanogaster*. *PLoS Genet*, **9**, e1003534.

Corbett-Detig RB, Zhou J, Clark AG, Hartl DL, Ayroles JF (2013) Genetic incompatibilities are widespread within species. *Nature*, **504**, 135–137.

Fiston-Lavier A-S, Singh ND, Lipatov M, Petrov DA (2010) *Drosophila melanogaster* recombination rate calculator. *Gene*, **463**, 18–20.

Kapun M, van Schalkwyk H, McAllister B, Flatt T, Schlötterer C (2013) Inference of chromosomal inversion dynamics from Pool-Seq data in natural and laboratory populations of *Drosophila melanogaster*. *Molecular Ecology*.

Kofler R, Schlötterer C (2013) A guide for the design of evolve and resequencing studies. *Molecular Biology and Evolution*, mst221.

Mackay TFC, Richards S, Stone EA *et al.* (2012) The *Drosophila melanogaster* Genetic Reference Panel. *Nature*, **482**, 173–178.

Tobler R, Franssen SU, Kofler R *et al.* (2013) Massive habitat-specific genomic response in *D. melanogaster populations* during experimental evolution in hot and cold environments. *Molecular Biology and Evolution*, mst205.
