# Supplementary figures and images for: Patterns of Linkage Disequilibrium and Long Range Hitchhiking in Evolving Experimental Drosophila melanogaster Populations
Source: Mol Biol Evol. 2014 Nov 17;32(2):495–509. doi: 10.1093/molbev/msu320 (PMC4298179; doi:10.1093/molbev/msu320)

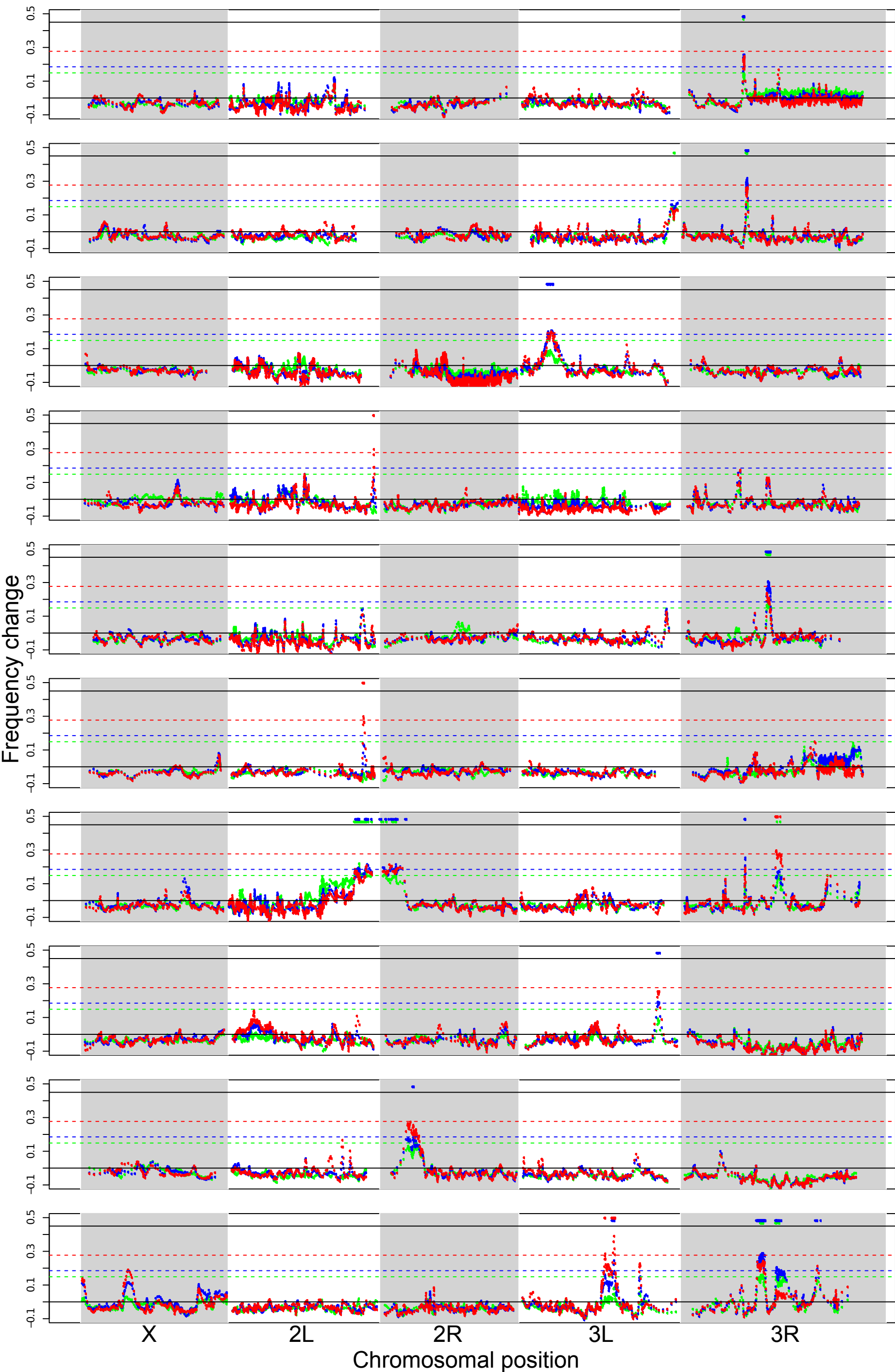

Supplement: Supplementary Data [file supp_msu320_Supplementary_material_figures-S10.pdf]
