# Supplementary material for: Patterns of Linkage Disequilibrium and Long Range Hitchhiking in Evolving Experimental Drosophila melanogaster Populations
Source: Mol Biol Evol. 2014 Nov 17;32(2):495–509. doi: 10.1093/molbev/msu320 (PMC4298179; doi:10.1093/molbev/msu320)

**Supplemental Material**

Figure S11 C-P: Visualization of each haplotype-block together with the evolved haplotypes from generation 67

C) haplotype-block 1


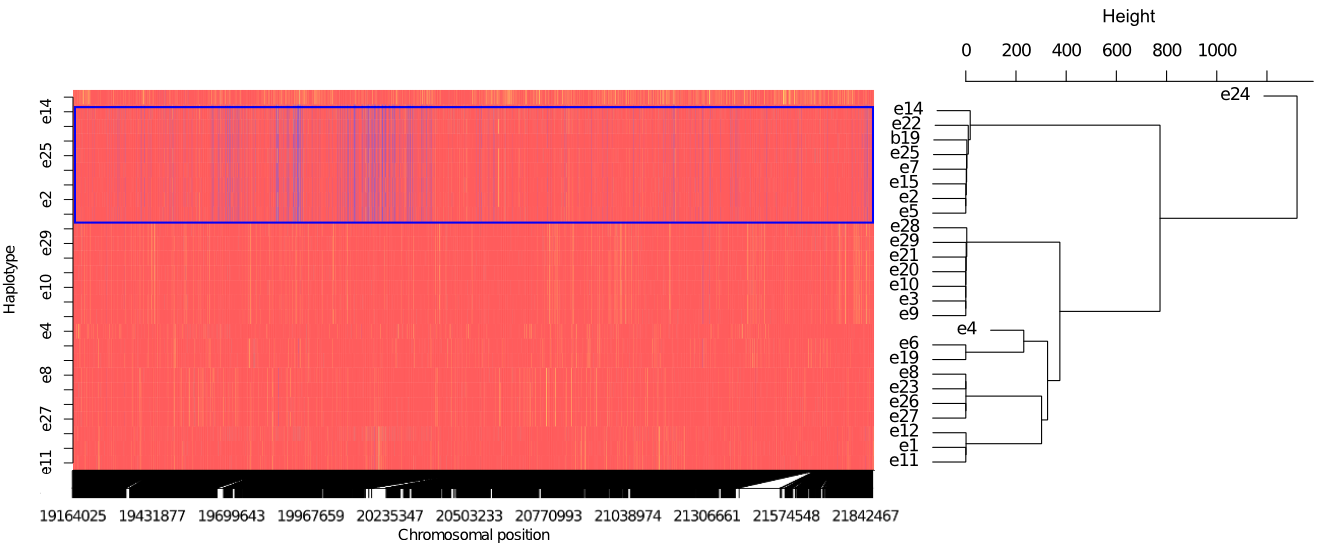


D) haplotype-block 2


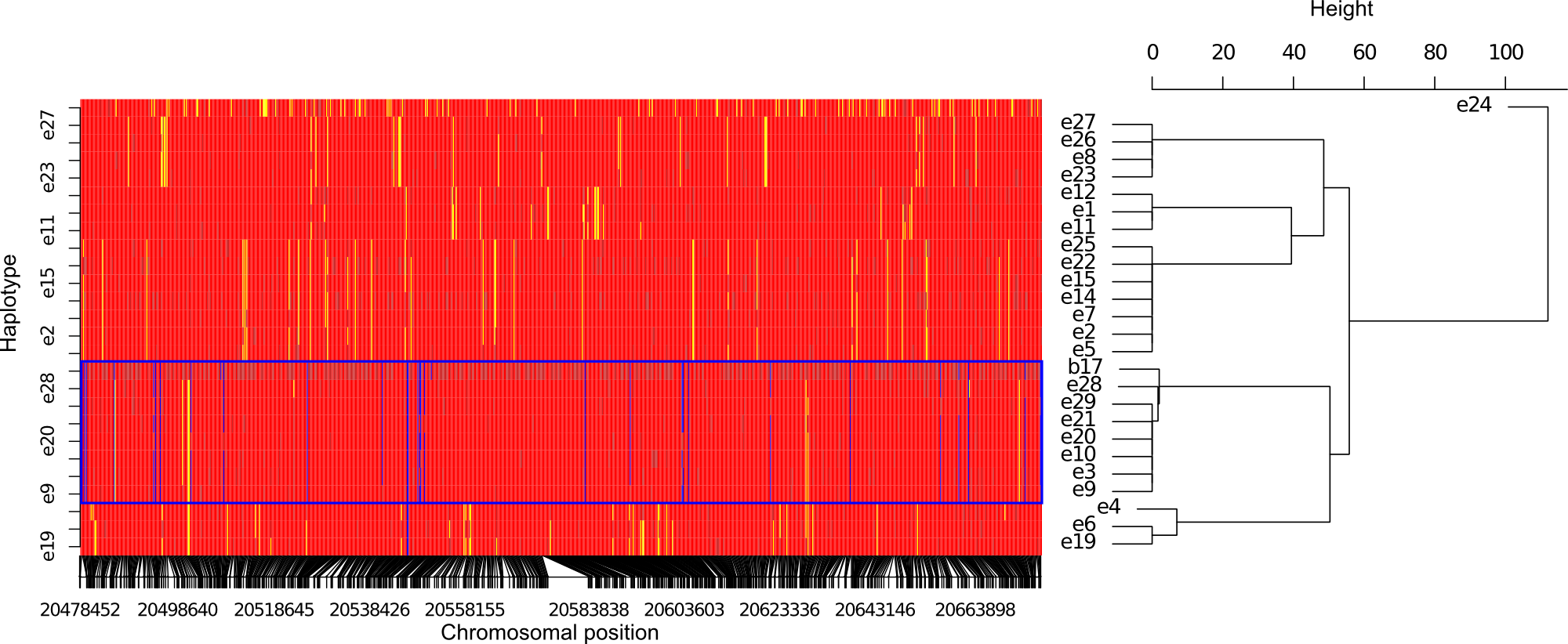


E) haplotype-block 3


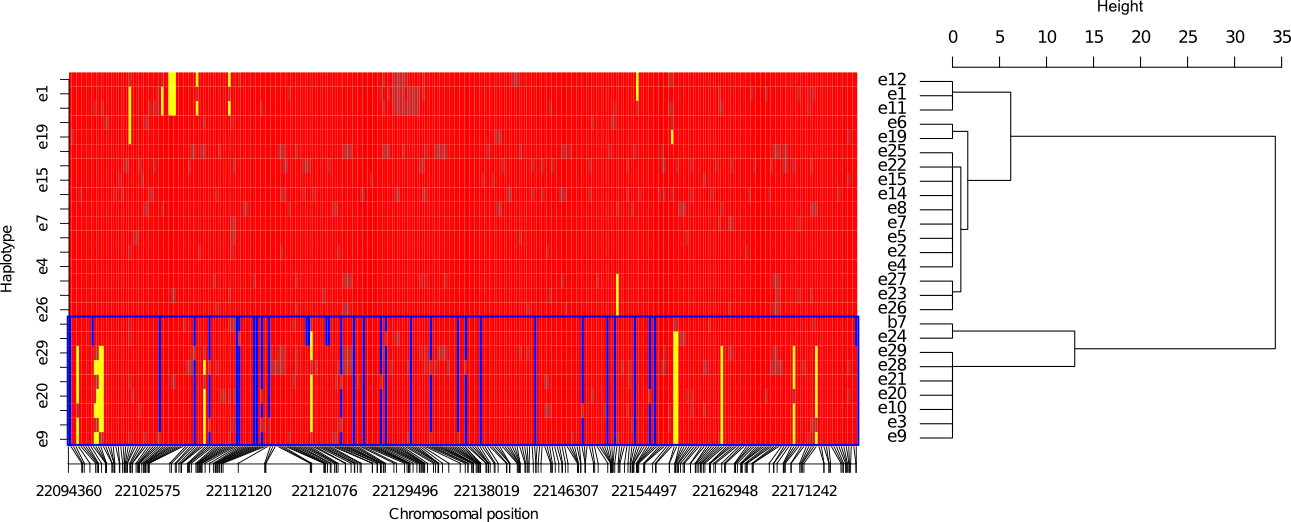


F) haplotype-block 4


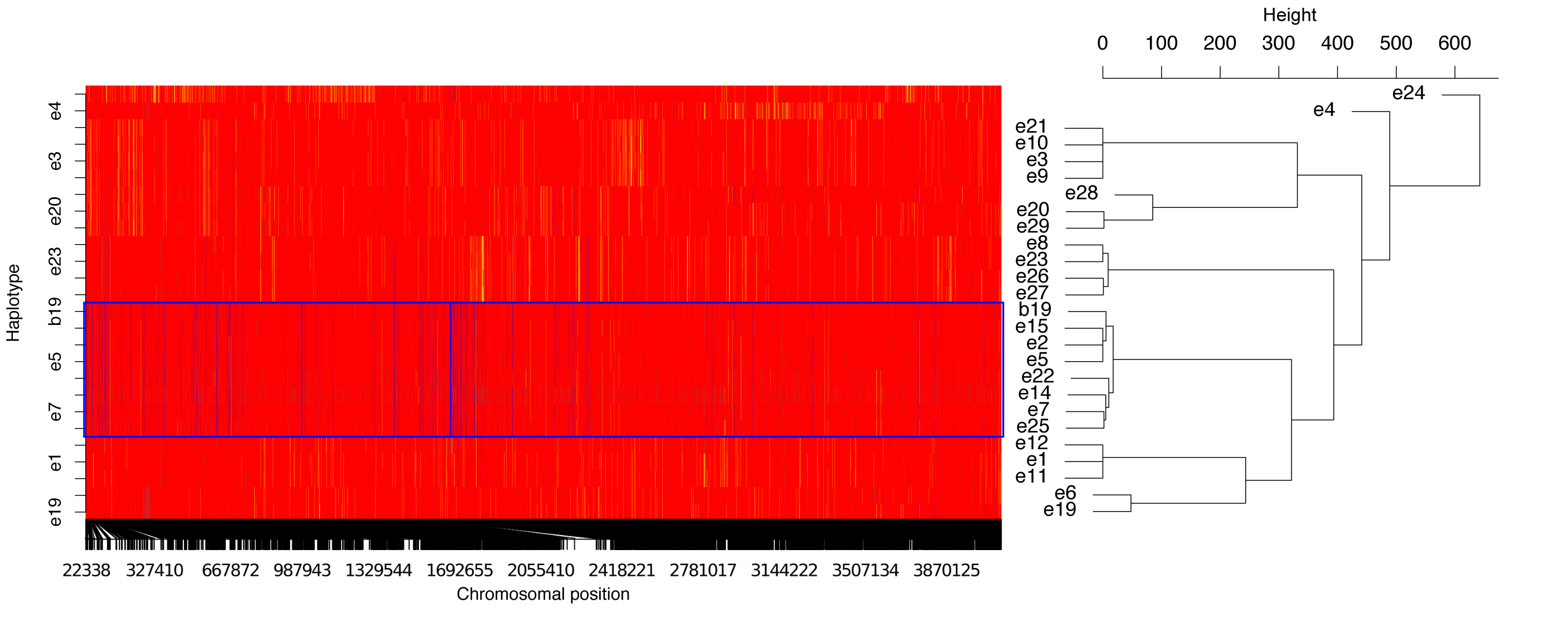


G) haplotype-block 5


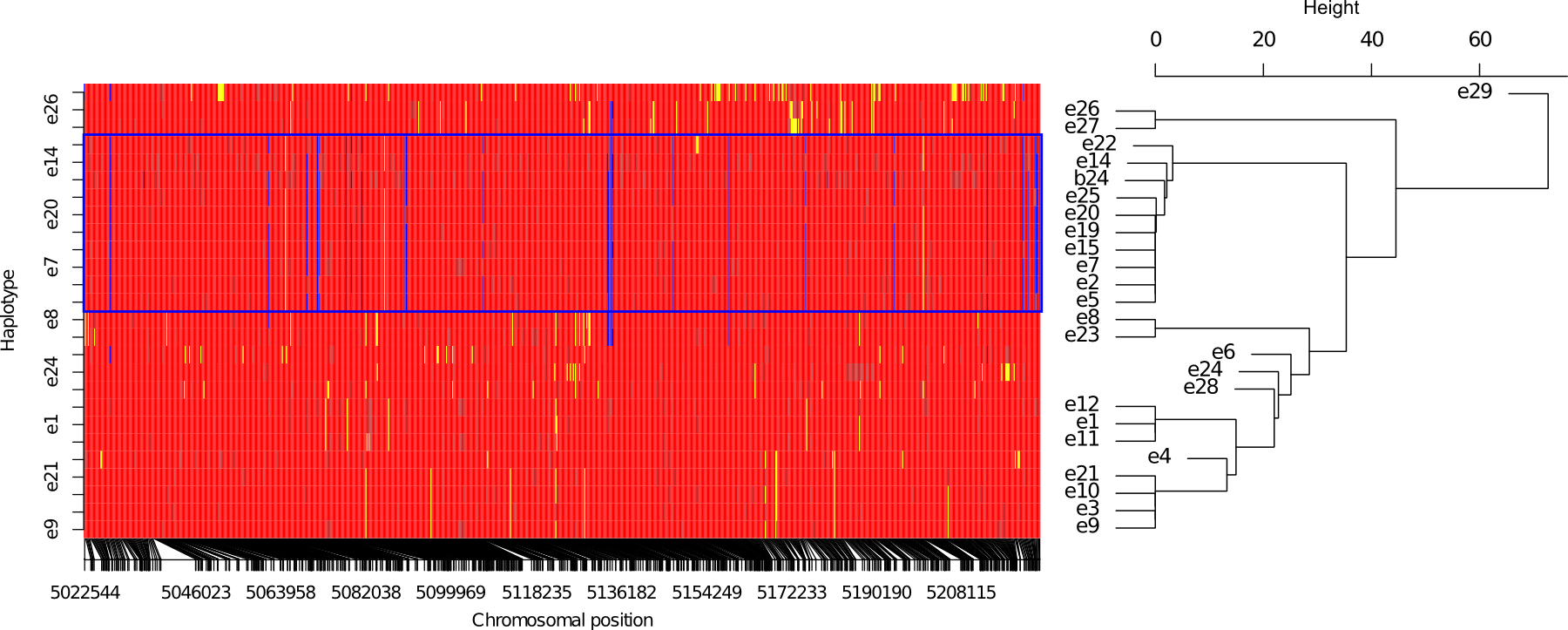


H) haplotype-block 6


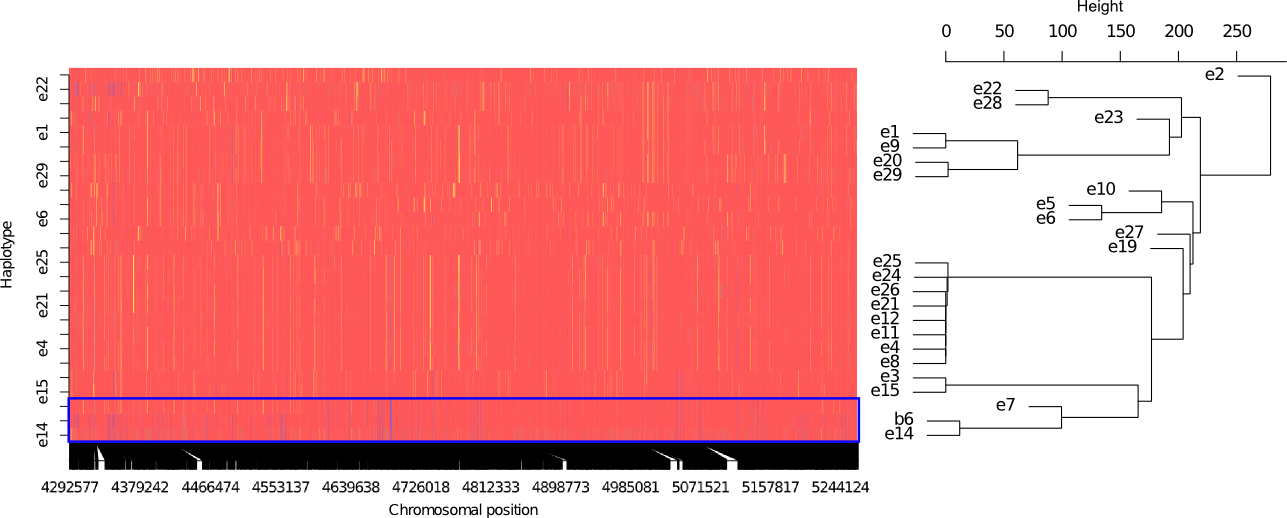


I) haplotype-block 7


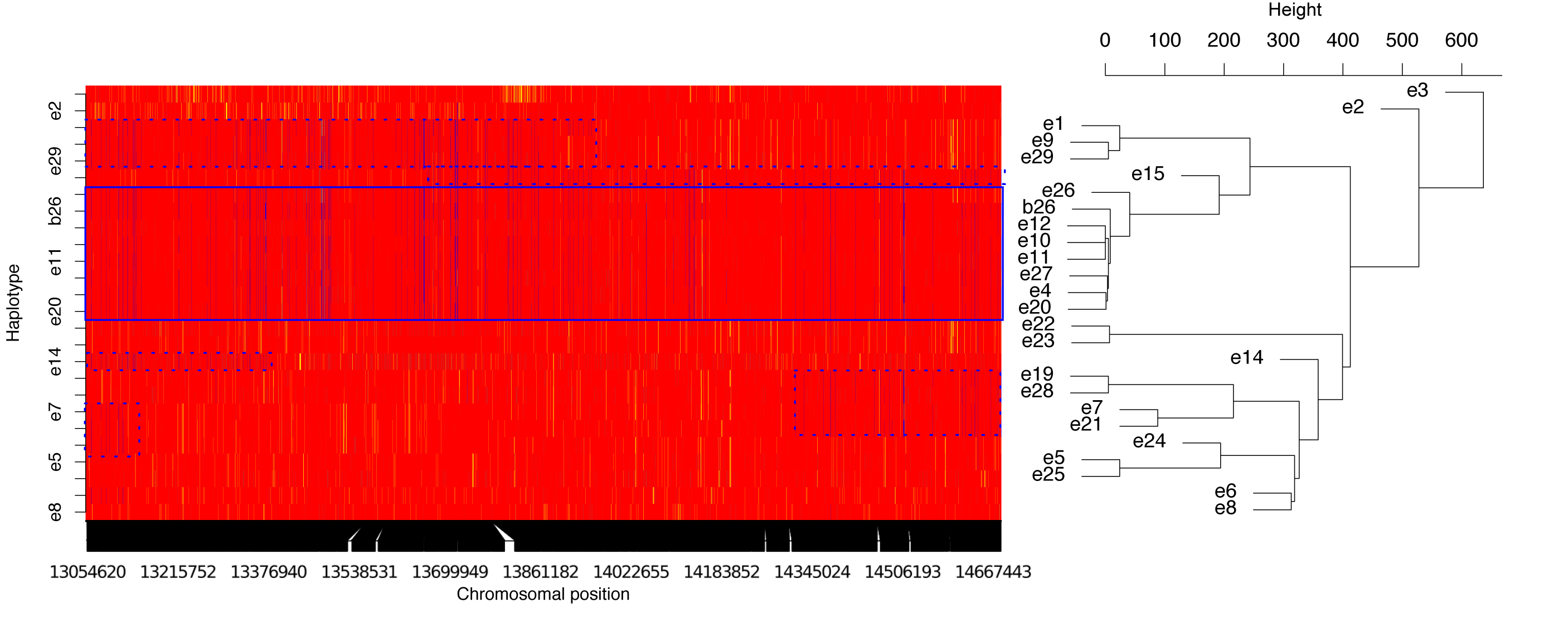


J) haplotype-block 8


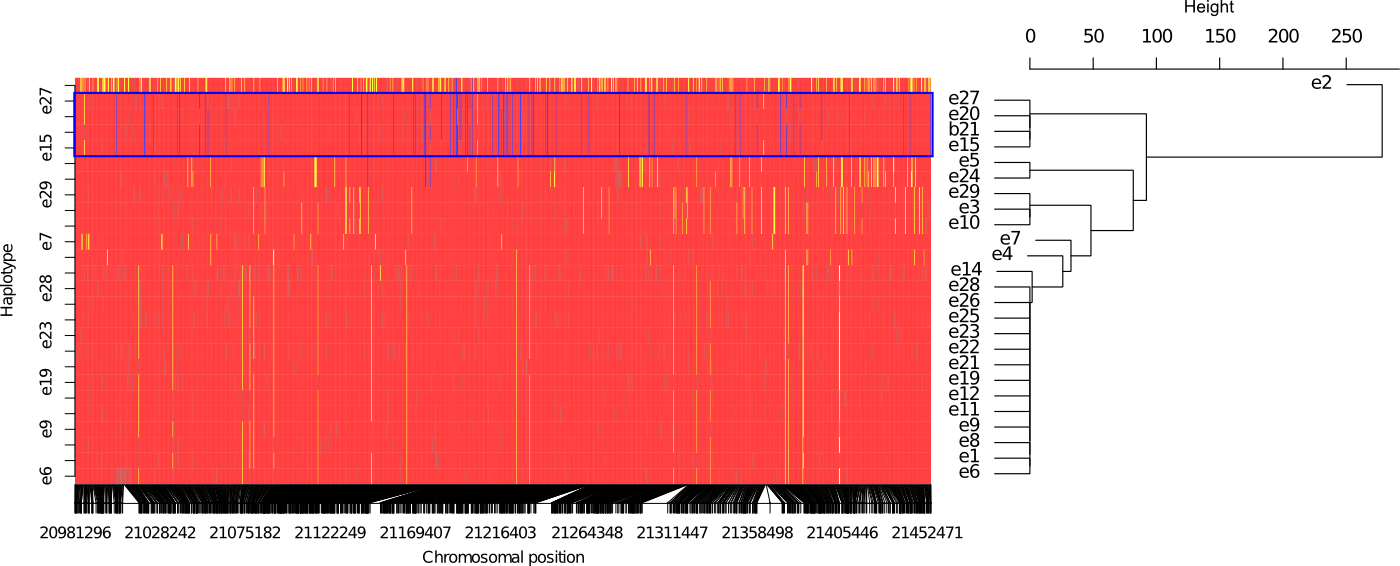


K) haplotype-block 10


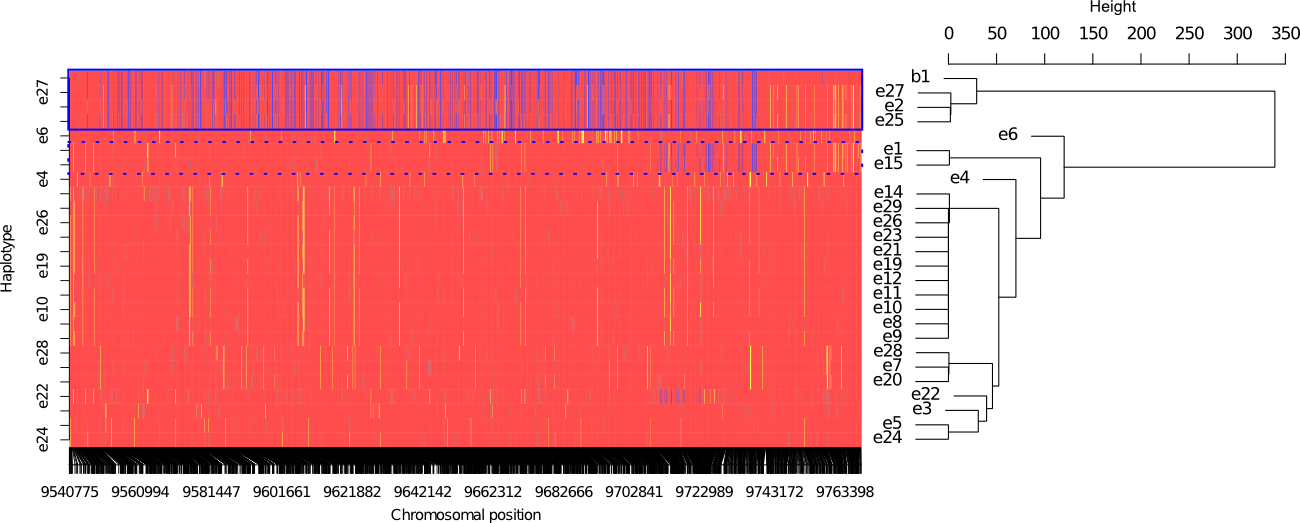


L) haplotype-block 13


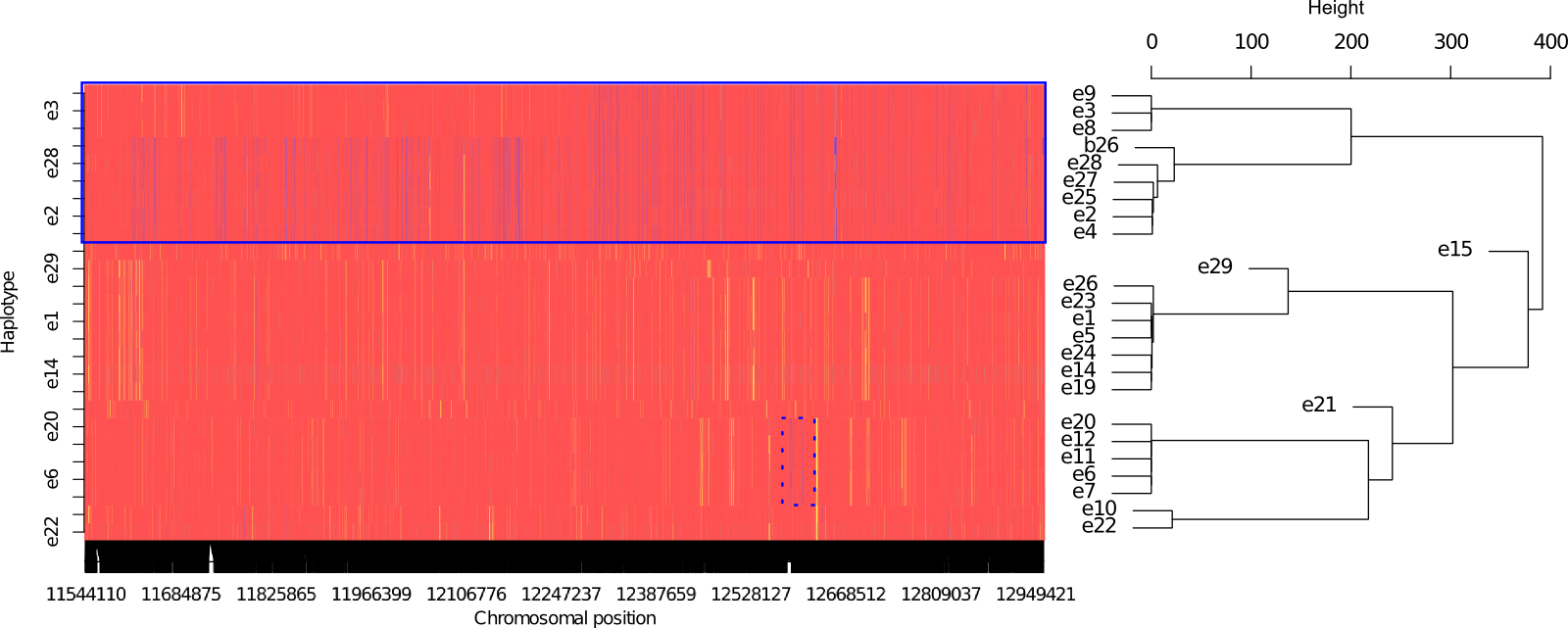


M) haplotype-block 14


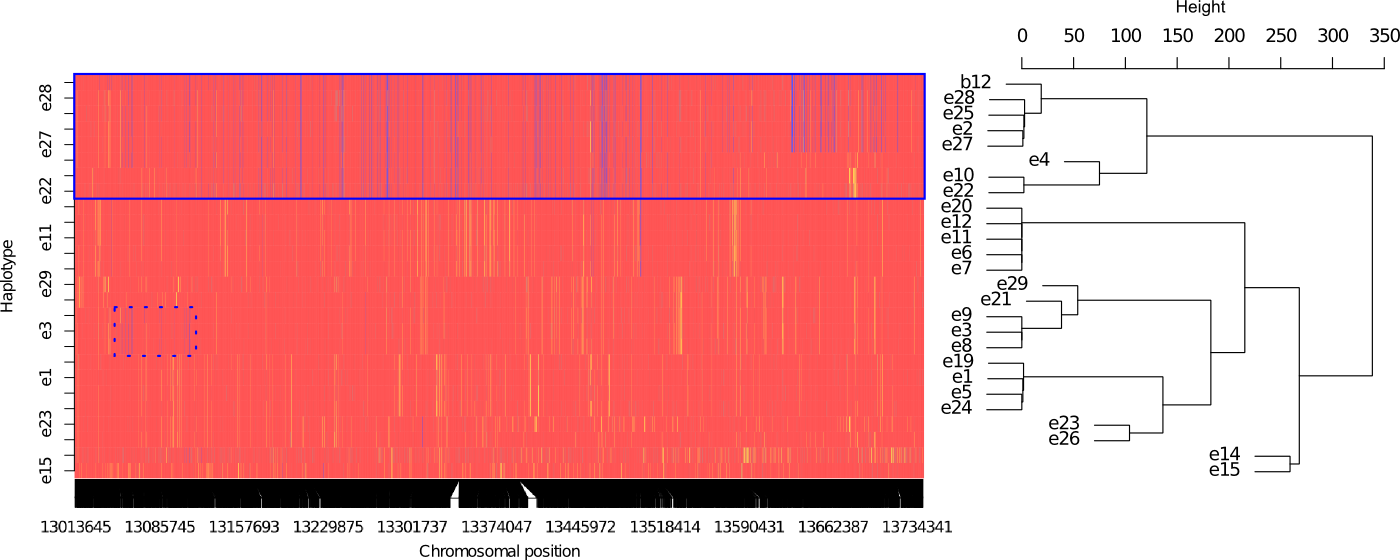


N) haplotype-block 15


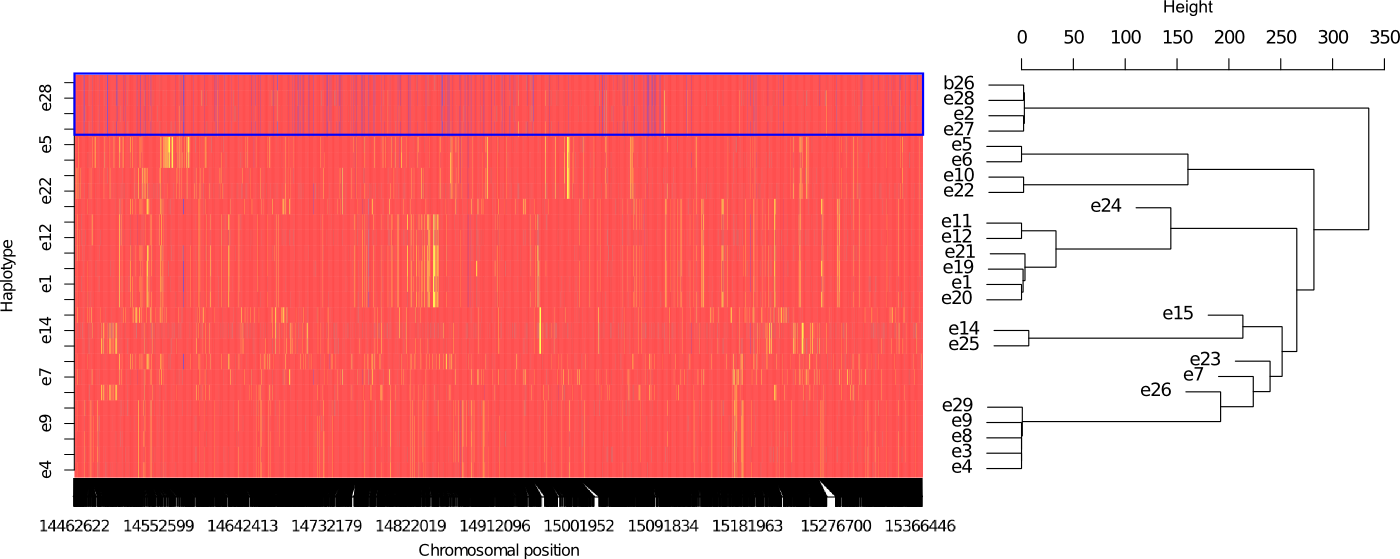


O) haplotype-block 16


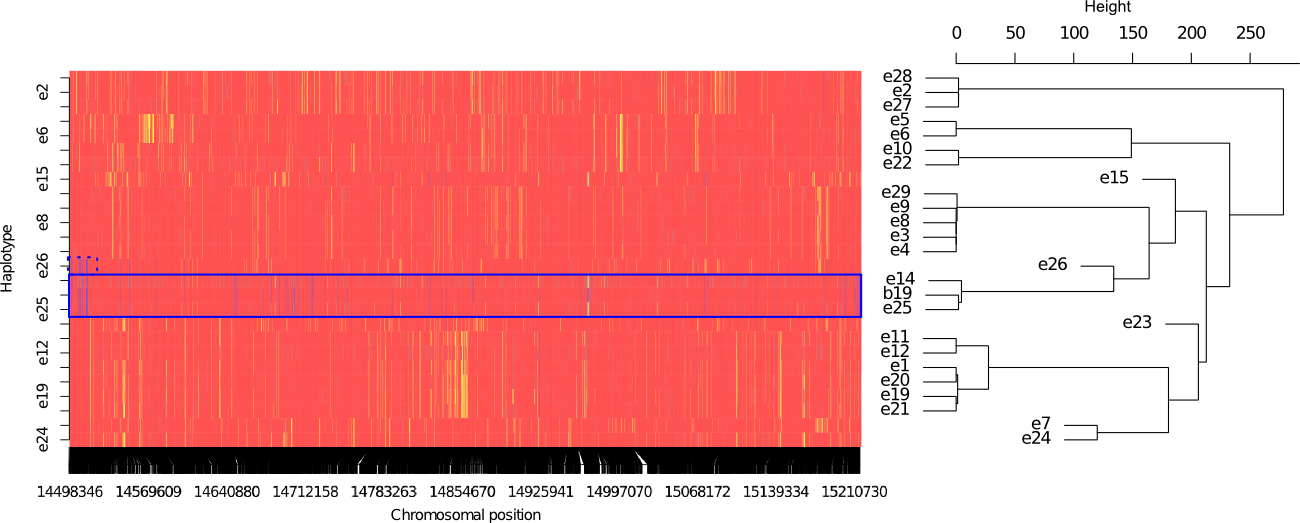


P) haplotype-block 17


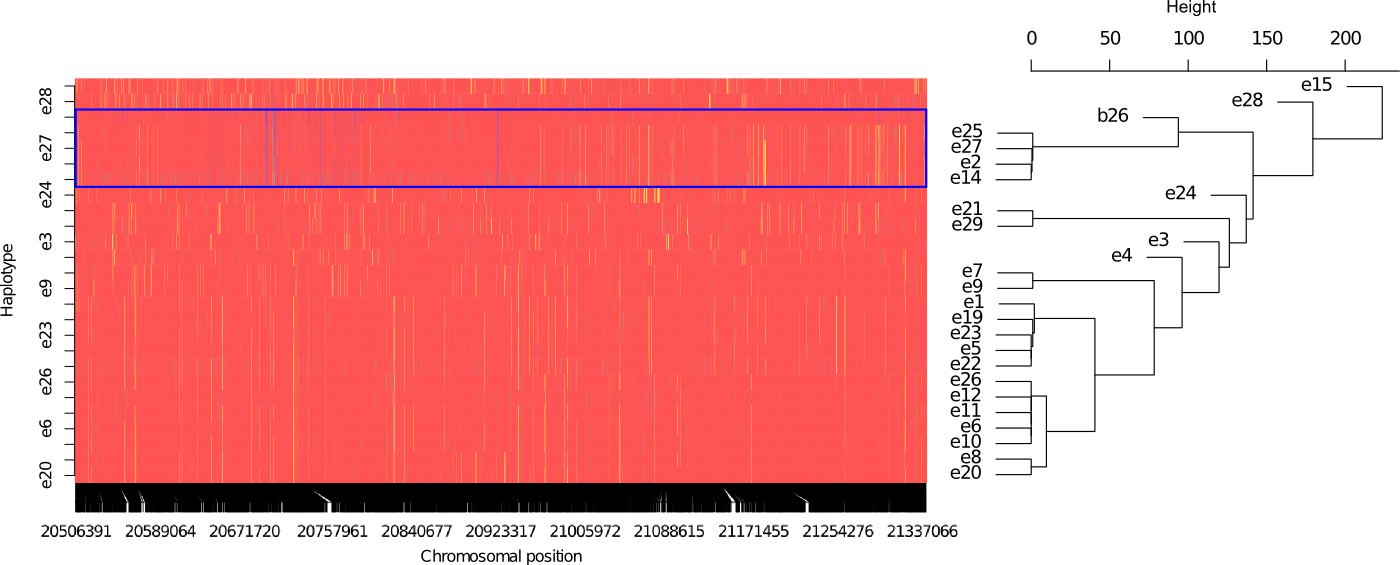

Supplement: Supplementary Data [file supp_msu320_Supplementary_material_figures-S11.doc]
